# RNA-seq Analysis Reveals That an ECF σ Factor, AcsS, Regulates Achromobactin Biosynthesis in *Pseudomonas syringae* pv. syringae B728a

**DOI:** 10.1371/journal.pone.0034804

**Published:** 2012-04-18

**Authors:** Jessica W. Greenwald, Charles J. Greenwald, Benjamin J. Philmus, Tadhg P. Begley, Dennis C. Gross

**Affiliations:** 1 Department of Plant Pathology and Microbiology, Texas A&M University, College Station, Texas, United States of America; 2 Department of Chemistry, Texas A&M University, College Station, Texas, United States of America; University of Padova, Medical School, Italy

## Abstract

Iron is an essential micronutrient for *Pseudomonas syringae* pv. syringae strain B728a and many other microorganisms; therefore, B728a has evolved methods of iron acquirement including the use of iron-chelating siderophores. In this study an extracytoplasmic function (ECF) sigma factor, AcsS, encoded within the achromobactin gene cluster is shown to be a major regulator of genes involved in the biosynthesis and secretion of this siderophore. However, production of achromobactin was not completely abrogated in the deletion mutant, implying that other regulators may be involved such as PvdS, the sigma factor that regulates pyoverdine biosynthesis. RNA-seq analysis identified 287 genes that are differentially expressed between the AcsS deletion mutant and the wild type strain. These genes are involved in iron response, secretion, extracellular polysaccharide production, and cell motility. Thus, the transcriptome analysis supports a role for AcsS in the regulation of achromobactin production and the potential activity of both AcsS and achromobactin in the plant-associated lifestyle of strain B728a.

## Introduction

Iron is the fourth most common element in the Earth's crust and, as such, has played an important role in microbial metabolism for millions of years [1]. However, in environments with physiological pH and oxygen tension, ferric iron (Fe^3+^) is oxidized and forms stable, insoluble ferric oxide hydrate complexes that cannot be readily utilized by microorganisms [2], [3]. The formation of these stable complexes leaves an environment with a free iron content of only 10^−9^ to 10^−18^ M, well below the 10^−6^ to 10^−8^ M required by most microorganisms [4], [3]. The ability to acquire and utilize iron is essential to the proper metabolism and basic cellular functioning of most macro and microorganisms [3]. In addition to functioning as a co-factor for many metabolic enzymes, iron plays a predominant role in the electron transfer chain, as well as, the catalysis of numerous cellular redox reactions [3]. Sufficient iron concentrations are also important for the biosynthesis of certain secondary metabolites. For example, the plant pathogenic bacterium *Pseudomonas syringae* pv. syringae strain B301D requires a minimum of 2 µmol/L available iron for the biosynthesis of the potent phytotoxins and major virulence factors, syringomycin and syringopeptin [5]. Thus, *P. syringae* utilizes high affinity iron uptake systems to procure iron, which is critical to its survival in the plant environment and expression of virulence determinants.

A method that microorganisms utilize to acquire sufficient iron from the environment is the biosynthesis of low molecular weight, chelating molecules called siderophores, meaning “iron carrier” in Greek [6]. These high-affinity iron-scavenging molecules compete with other microorganisms and host molecules for environmentally available iron resources [7]. The number and type of siderophores utilized by plant-associated bacteria is not uniform and frequently varies from species to species, and even between strains [8].

For some plant pathogenic bacteria, such as the vascular wilt and soft rot pathogen *Dickeya dadantii* strain 3937, siderophores serve as major virulence factors [9], [10]. *Dickeya dadantii* strain 3937 produces two siderophores with distinct iron binding capacities, chrysobactin and achromobactin, both of which are necessary for bacterial growth in planta and subsequent plant disease [9], [10]. Achromobactin is utilized at the establishment of bacterial growth in the plant, since it is produced at less stringent iron limitation levels than the siderophore chrysobactin [9], [8]. As the in planta bacterial population rises, the available iron becomes increasingly depleted and the *Dickeya* population gradually increases production of chrysobactin, which has a higher binding affinity for iron [9], [8]. Hence, *Dickeya dadantii* strain 3937 capitalizes on the unique iron binding capacities of the two siderophore systems and utilizes the systems concurrently as a method of adapting to changing iron availability in the plant environment [9], [8].

Alternatively, *P. syringae* pv. tomato strain DC3000, the causal agent of bacterial speck on tomato, does not require siderophore biosynthesis for pathogenicity or virulence [7]. A recent study by Jones and Wildermuth [7] concluded that DC3000 is capable of causing disease despite the loss of its three siderophore systems, pyoverdine, yersiniabactin, and citrate. This suggests that DC3000 is able to import sufficient quantities of iron from the apoplastic spaces of infected plants, without the use of high-affinity iron binding siderophore molecules [7].

*P. syringae* pv. syringae strain B728a, the causal agent of brown spot on bean (*Phaseolus vulgaris* L.), is distinct from *P. syringae* pv. tomato DC3000 in both lifestyle and host range [11]. While both pathogens invade the apoplastic spaces of plant tissue to cause disease, B728a is also an exceptionally efficient epiphyte and can establish populations on the leaf surfaces of >10^7^ cells per gram [11]. Like all fluorescent pseudomonads, both B728a and DC3000 utilize the non-ribosomal peptide synthetase (NRPS) assembled siderophore, pyoverdine [12]. However, B728a also encodes an NRPS-independent siderophore (NIS) synthetase system for synthesis of the citrate derivative achromobactin [12], [11], [13]. The siderophores produced by B728a are identical to those utilized by the soybean epiphyte *P. syringae* pv. syringae strain 22d/93 [8]. The epiphytic fitness of strain 22d/93 was significantly impaired by disruption of either pyoverdine or achromobactin biosynthesis; thereby implicating that these iron-scavenging molecules may be important for the epiphytic survival and fitness of B728a rather than serving as a virulence factor [8].

In iron depleted environments it is essential that bacteria are able to quickly adapt. The expression of iron-related genes, including siderophore biosynthesis and transport genes, requires RNA-polymerase σ-factors from the extracytoplasmic function (ECF) subfamily of the σ^70^ family, which typically respond to environmental signals [14]. Herein we evaluate the regulon of an uncharacterized σ^70^ factor encoded within the 14-gene cluster of B728a associated with the biosynthesis and transport of the siderophore achromobactin [13], [11]. By identifying gene targets regulated concurrently with achromobactin biosynthesis and secretion, we aimed to understand more about when and how achromobactin is utilized in the B728a lifecycle. In this study it is demonstrated that the σ^70^ factor, AcsS, regulates the biosynthesis and secretion of achromobactin, as well as, other B728a genes associated with epiphytic growth and survival.

## Materials and Methods

### Bioinformatic analyses

Genome database searches were performed with the Basic Local Alignment Search Tool (BLAST) at NCBI (http://blast.ncbi.nlm.nih.gov/Blast.cgi).

### Bacterial strains, plasmids, and growth conditions

The bacterial strains and plasmids used in this study are listed in Table 1. For general cloning *Escherichia coli* DH10B was cultured in Luria-Bertani (LB) liquid or agar medium at 37°C [15], [16]. For topoisomerase reactions One Shot® TOP10 *Escherichia coli* or Mach 1 T1™ *Escherichia coli* cells were used in accordance with the manufacturer's protocol (Invitrogen, Carlsbad, CA). *P. syringae* pv. syringae B728a and mutant derivatives were grown routinely at 26°C with shaking at 200 rotations per minute (rpm) in King's B [17] and LB media for general growth. A Hrp-inducing minimal media (HMM) [0.2 M KH_2_PO_4_, 1.2 M K_2_HPO_4_, 1.3 M (NH_4_)_2_SO_4_, 5.9 M MgCl_2_, 5.8 M NaCl, 0.2% fructose] was used for iron-limited culture conditions [18]. Water for use in iron-limited media was treated to remove free iron using 10 grams of Chelex®100 (Bio-Rad) per 100 ml of ultrapure water (Barnstead E-Pure D4642-33). Antibiotics were added as needed at the following concentrations (µg ml^−1^): rifampicin 100, kanamycin 75, tetracycline 20, chloramphenicol 20, gentamicin 5, and spectinomycin 100.

**Table 1:** Strains and plasmids used in this study.

Designation	Relevant characteristics	Source
Bacterial strains		
*E. coli*		
DB3.1	F^−^ *gyrA462 endA1 glnV44* Δ(*sr1-recA*) *mcrB mrr hsdS20*(rB^−^ mB^−^) *ara14 galK2 lacY1 proA rpsL20*(Sm^r^) *xyl5* Δ*leu mtl1*	[56]
DH10B	Δ(mrr-*hsdRMS-mcrB*) *deoR recA1 endA1 araD139* Δ(*ara*, *leu*)7697 *galU galK λ^−^ rpsL nupG*	[57]
One Shot®TOP10	F^−^ *mcrA* Δ(mrr-hsdRMS-mcrB) Φ80*lacZΔM15* Δ*lacX74 recA1 araD139* Δ*(ara-leu)7697 galU galK rpsL (Str^R^) endA1 nupG*	Invitrogen
Mach1 T1™	F^−^ Δ*recA1398 endA1 tonA* φ80(*lacZ*)ΔM15 Δ*lacX74 hsdR*(rK^−^ mK^+^)	Invitrogen
SW105	DY380 (*cro*-*bioA*) <>*araC*-P_BAD_ *Cre* Δ*galK*	National Cancer Institute
*P. syringae* pv. syringae		
B728a	Wild-type, bean pathogen; Rif^r^	[58]
B728aΔ*acsS*	*acsS* mutant derivative of B728a, Rif^r^	This study
B728a ADB1005 (PVD^−^)	*pvdL::nptII* (Kan^r^)	[13]
B728aΔ*gacS*	*gacS* mutant derivative of B728a, Rif^r^	[21]
B728a *ΔacsS* pPROBE-KT′: *acsS*	Complemented strain of the *acsS* mutant derivative of B728a, Rif^r^ Km^r^	This study
Plasmids		
pBH474	*flp* constitutively expressed; Gm^r^ Suc^s^	[59]
pENTR/D-TOPO	Gateway entry vector; Km^r^	Invitrogen
pE*acsS*	pENTR/D-TOPO carrying *acsS*, Km^r^	This study
pKD13	Template plasmid containing FRT-flanked *nptII*	[55]
pLVCD	Gateway destination vector for mating with *P. syringae*; pBR322 derivative with *mob* genes from RSF1010; Tc^r^ Ap^r^ Cm^r^	[51]
pLV*acsS*	pLVCD carrying *acsS*; Tc^r^ Ap^r^	This study
pLV*acsS*-FP	pLVCD carrying upstream and downstream regions of *acsS* fused to *nptII*; Tc^r^ Ap^r^ Km^r^	This study
pRK2073	Helper plasmid; Sp^r^ Trm^r^	[22]

For iron-limited conditions, glassware was treated to remove exogenous iron as described by Kadurugamuwa et al. [19]. Glassware was soaked in 5% Extran MA01 (EMD Chemicals, Germany) for 6 hours followed by soaking in 0.01% EDTA (J.T. Baker, Phillipsburg, NJ) for 12 hours. Glassware was rinsed in 1% HCl, followed by extensive rinsing in ultrapure water (Barnstead E-Pure D4642-33). Glassware was dried in a 160°C oven for 3 hours, followed by autoclaving.

### Construction of markerless deletion mutations in B728a

Targeted deletion mutants in B728a were made using a modified version of the phage lambda Red recombinase system developed by Datsenko and Wanner [20], [21]. With this strategy, the gene of interest (GOI) along with 3 to 4 kb of flanking DNA on each side was PCR amplified using Phusion® high fidelity, long-range proofreading polymerase (ThermoScientific F-553S) (flank-GOI-flank). The primers for this PCR reaction, Prr2580F and Prr2580R, were designed to add a TOPO cloning tag onto the 5′ end of the PCR product (Table 2). The purified PCR product was then transferred into the Gateway entry vector pENTR/D-TOPO (Invitrogen pENTR/D-TOPO cloning kit catalog #45-0218) and transformed into chemically competent *E. coli* Mach 1 cells (pENTR: flank-GOI-flank). The gene of interest with its flanking regions was recombined into the *Pseudomonas* suicide vector, pLVC-D [21], using a Gateway reaction (Invitrogen LR Clonase II catalog #11791-020). Site-specific recombination proteins from the bacteriophage lambda were used to recombine the gene of interest and flanking region from the pENTR vector into the pLVC-D destination vector (pLVC-D: flank-GOI-flank).

**Table 2:** Primers used for PCR and qRT-PCR amplification.

Name	Sequence	Source
Prr2580F	CACCAAAAACGCCCGCTGATGAG	This study
Prr2580R	GCGCCGGCCGAACTCCA	This study
Prr2580KmF	CTCATTTGGCGCATCCCATCGCTCAAGGCTTTTGACGTGTAGGCTGGAGCTGCTTCG	This study
Prr2580KmR	CTACCTCGCGTTCTTCGTTCGAAGGTGCCGGGCGAAATTCCGGGGATCCGTCGACC	This study
qRTrecAF	CTTCGGTACGCCTGGACA	[26]
qRTrecAR	AACTCGGCCTGACGGAAC	[26]
qRT16SF	ACACCGCCCGTCACACCA	[26]
qRT16SR	GTTCCCCTACGGCTACCTT	[26]
qRT2580F	ATCATCATGGCTGGCTGGAAAGC	This study
qRT2580R	TCAAGGCTGCGACGAGTGTAGAAA	This study

The pLVC-D: flank-GOI-flank destination vector was then transformed into the recombineering (recombination-mediated genetic engineering) strain *E. coli* SW105 (http://recombineering.ncifcrf.gov/). The genome of this strain contains a defective lambda prophage containing the Red recombinase genes. These genes are regulated by a temperature sensitive repressor, *cI857*, which is active at 32°C thereby preventing any recombination proteins from being produced. A 15 minute heat-shock at 42°C inactivates the *cI857* repressor and allows transcription of the Red recombinase proteins, which insert the linear DNA directly into the target site within the GOI. The linear DNA was a PCR product containing 36 bp of DNA flanking each side of the gene of interest and a kanamycin (Km) resistance cassette (amplified from the vector pKD13 using primers Prr2580KmF and Prr2580KmR) (Table 2). The pKD13 vector was used to amplify the FRT (FLP recognition target) sites for removal of the Km cassette. The linear DNA was recombined into the destination plasmid by electroporation of heat-shocked *E.coli* SW105: pLVC-D: flank-GOI-flank with the linear DNA product. The resulting *E.coli* SW105: pLVC-D: flank-Km-flank was triparentally mated with B728a to introduce the Km cassette in place of the gene of interest. The Km cassette was removed using FLP recombinase. Colony PCR and Southern blot analysis were used to confirm all double recombination mating events.

### General DNA manipulations

Restriction enzymes, Calf Intestinal Phosphatase (CIP), and T4 DNA ligase were purchased from New England Biolabs (Beverly, Mass.) and used according to the manufacturers' protocols. Thermo Scientific Phusion High-Fidelity DNA polymerase was purchased from Fisher Scientific. Cloning strategies for the amplification of target genes via PCR and utilization of Gateway technology were done in accordance with the manufacturer's protocols (Invitrogen) [22]. Recombination between pENTR constructs and Gateway destination vectors were performed in accordance with the manufacturer's instructions provided for LR clonase (Invitrogen). Plasmids were incorporated into *E.coli* via chemical transformation or electroporation [16]. Tri-parental mating with the helper plasmid pRK2073 was used for the incorporation of plasmids in B728a for recombination events [23]. Primer sequences are listed in Table 2 and standard PCR cycling conditions were used.

### Quantitative real time PCR

Quantitative real time reverse-transcription PCR (qRT-PCR) was used to determine if the sigma factor gene, *acsS*, is responsive to low environmental iron conditions. Experimental design and controls were based on the guidelines outlined by Bustin et al. [24]. Total RNA samples were extracted from B728a that was grown to late logarithmic phase (OD_600_ of 0.6) at 26°C in iron-limited HMM media, HMM media plus 10 µM iron, and HMM media plus 100 µM iron. Three biological replicates were performed for each media condition, with each biological replicate performed on a separate day. The cultures were fixed using RNA protect™ Bacterial Reagent (Qiagen), in a ratio of 2 ml of reagent per 1 ml of bacterial culture. Centrifugation was used to pellet the cells (5000 rpm, 4°C, 20 min) and the supernatant was discarded. Cell lysis was performed using 7 mg ml^−1^ lysozyme (M.P. Biomedicals) in TE buffer (10 mM TrisCl, 1 mM EDTA, pH 8.0) with frequent vortexing for 7 min at room temperature. Samples were extracted from B728a using an RNeasy® Mini Kit (Qiagen) and eluted in RNase/DNase free water. RNA samples were treated with TURBO™ DNase (Ambion®) using the manufacturer's protocol. The RNA was tested for DNA contamination using qRT-PCR in which the RNA was used as the template and the reverse transcription reaction is not performed. The RNA quality was measured at the Texas AgriLife Genomics and Bioinformatics Services using an Agilent 2100 Bioanalyzer (Agilent Technologies, Inc.) and only RNA samples with an RNA Integrity Number (RIN) above 8.0 were selected [25]. Total RNA samples were quantified using micro-spectrophotometry (Nano-Drop Technologies, Inc.).

Total RNA (150 ng per biological sample) was converted to double stranded cDNA by reverse transcription using Super Script Vilo™ cDNA Synthesis kit (Invitrogen™). Reverse transcription was conducted with the following temperature cycle: 10 min at 25°C, 60 min at 42°C, 5 min at 85°C. The double stranded cDNA was quantified using micro-spectrophotometry (Nano-Drop Technologies, Inc.) and samples were diluted to 10 ng µl^−1^.

qRT-PCR was performed using an Applied Biosystems 7500 Fast Real-Time PCR System with the SYBR® GreenER™ Reagent System (Invitrogen™). For each 20 µl reaction the following was used: 10 µl SYBR® GreenER™ qPCR SuperMix Universal, 8.16 µl nuclease free water, 0.04 µl ROX reference dye, 0.4 µl forward primer (200 nM final), 0.4 µl reverse primer (200 nM final), 1 µl template DNA (10 ng µl^−1^). Primers used for these reactions are listed in Table 2, with the primers qRTrecAF/qRTrecAR and qRT16SF/qRT16SR being utilized for normalization [26]. Analysis of the dissociation curve ensured that a single product was amplified; this cycle consisted of 95°C for 15 sec, 60°C for 1 min, 95°C for 15 sec. All primer pairs amplified a single product in the conditions tested. The linearity of detection was confirmed for each primer pair by measuring a five-fold dilution curve for cDNA synthesized from total RNA in the conditions tested in this study. The correlation coefficient for this dilution curve was evaluated and confirmed to be at least 0.98 (r^2^>0.98). The efficiency of the primers was calculated using the slope of the line from the five-fold dilution curve using the following equation: Efficiency = 10^(−1/slope)^−1. Only primers with efficiencies between 90% and 110% were used.

Data was analyzed using the comparative C_t_ method, wherein the C_t_ values of the samples of interest are compared to the C_t_ values of a control. All the C_t_ values were normalized to endogenously expressed genes, in this case the *recA* housekeeping gene and a 16 S ribosomal RNA gene [26]. The ΔC_t_ was calculated as: C_t sample_−C_t housekeeping_. The ΔΔC_t_ was the fold change between the control sample and the sample of interest and was calculated as: ΔC_t sample of interest_−ΔC_t control sample_.

### Preparation of RNA samples for transcriptome analyses

RNA sample preparation and cDNA library generation was performed according to procedures outlined by Croucher et al. [27] and Perkins et al. [28] with slight modifications. RNA samples were extracted from B728a and B728a Δ*acsS* grown to late logarithmic phase (OD_600_ of 0.6) in iron-limited HMM media at 26°C, shaking at 200 rpm. The starting OD_600_ for each culture was less than 0.01. The three biological replicates of each strain were extracted on separate days with separate batches of media. The cultures were fixed using RNA protect™ Bacterial Reagent (Qiagen), in a ratio of 2 ml of reagent per 1 ml of bacterial culture. Centrifugation was used to pellet the cells (5000 rpm, 4°C, 20 min) and the supernatant was discarded. Cell lysis, RNA extraction, DNase treatment, and RNA quality analysis were performed as described above. The RNA was tested for DNA contamination using quantitative Real-Time reverse-transcription PCR (qRT-PCR) in which the RNA was used as the template and reverse transcription reaction was not performed. The RNA quality was measured using an Agilent 2100 Bioanalyzer and only RNA samples with an RNA Integrity Number (RIN) above 8.0 were selected [25]. Total RNA samples were quantified using micro-spectrophotometry (Nano-Drop Technologies, Inc.) and the Quant-iT™ RiboGreen® RNA assay kit (Invitrogen).

### RNA sample processing and reverse transcription

Complementary oligonucleotide hybridization was utilized to remove the 16 S and 23 S rRNA using the MICROBExpress™ bacterial mRNA enrichment kit (Ambion®) with *Pseudomonas* specific hybridization sequences provided by the manufacturer. Denaturing gel electrophoresis was used to ensure the removal of rRNA; a 2% Low Range UltraPure™ agarose denaturing gel was prepared with Tris-acetate-EDTA (TAE). Samples were prepared in a denaturing loading dye with a final concentration of 20 mM EDTA and were denatured at 95°C for 10 min prior to being loaded on the gel. The gel electrophoresis was performed using 4× TAE buffer and run at 60 V. Enriched mRNA samples were quantified using micro-spectrophotometry and equal quantities of three biological samples were pooled to form a single mRNA sample for both B728a and B728a Δ*acsS* with a total concentration of 1.8 µg for each sample [29], [30]. The pooled mRNA samples were denatured at 65°C for 5 min with 50 ng µl^−1^ random hexamer primers (Invitrogen™) and 10 mM dNTPs and placed on ice for 5 min. SuperScript III (Invitrogen™) reverse transcriptase was used to synthesize single stranded cDNA using the manufacturer's protocol, the synthesis cycle was 10 min at 25°C, 180 min at 45°C, 15 min at 70°C, 5 min at 85°C. *E. coli* RNase H (Invitrogen™) was used to remove complementary RNA. The single stranded cDNA was purified using the Wizard® SV Gel and PCR Clean-Up System (Promega) and quantified using micro-spectrophotometry.

### Library construction

Sequencing libraries for the Illumina GAIIX platform were constructed as follows. A 5 µg aliquant of each single stranded cDNA sample was diluted in TE buffer to a total volume of 120 µl for cDNA shearing. The Texas AgriLife Genomics and Bioinformatics Services performed the cDNA shearing using the Covaris S2 Adaptive Focused Acoustic Disruptor with a 200 base pair fragmentation cycle (Run at 10%, Intensity: 5, 200 cycles per burst, 4 cycles of 60 sec each). The sheared cDNA was purified using the QIAquick PCR Purification Kit (Qiagen) and eluted in 30 µl buffer EB (10 mM TrisCl, pH 8.5) (Qiagen). Illumina DNA library construction was performed using the Illumina Paired-End Sequencing method (Cat. No. PE-102-1001) in accordance with the manufacturer's recommended protocol. After the ligation of Illumina adaptors, the samples were run on a denaturing gel (described above) and the band correlating to 200–250 base pairs on the denatured DNA ladder was selected. The selected DNA constructs were amplified by 18 cycles of PCR using the Phusion® high-fidelity DNA polymerase and primers PE 1.0 and PE 2.0 provided in the Illumina library kit. The amplified constructs were purified using the Wizard® SV Gel and PCR Clean-Up System (Promega) and quantified using micro-spectrophotometry (Nano-Drop Technologies, Inc.).

### Illumina library sequencing

Library quality and quantity was confirmed using an Agilent 2100 Bioanalyzer. Sequencing of the libraries was performed using an Illumina GAIIX at the Lerner Research Institue (Cleveland, OH). Each library was loaded onto a single lane of an Illumina GAIIX flow cell and single-end, 36-cycle sequencing was performed with all cluster formation, primer hybridization, and sequencing reactions in accordance with the manufacturer's recommended protocol.

### Read mapping to reference genome

Sequencing reads were mapped to the reference genome, *P. syringae* pv. syringae B728a (Genbank CP000075.1). The removal of sequence adapters, mapping to reference genome, and the normalization of gene expression was performed using CLC Genomics Workbench (V4.5, CLC Bio.). The normalization of gene expression by Reads per Kilobase per Million Mapped Reads (RPKM) was calculated using the methods described by Mortazavi et al. [31].

### Differential gene expression analysis

The differential gene expression of the pooled samples from each condition was analyzed using the R sequence package DEGseq, under the random sampling model using the RPKM values obtained from the previous step [32].

### Purification of achromobactin from B728a

Achromobactin was purified from B728a, B728a ADB1005 (PVD-) [13], and B728a Δ*acsS*. The purification procedure was modified from Berti and Thomas [13]. Cultures of the *Pseudomonas* strains were grown to late log phase (OD_600_ of 0.6). One milliliter of the culture was pelleted and washed three times in iron-limited HMM media. Cell pellets were re-suspended in 1 ml of iron-limited HMM media and used to inoculate 2800 ml flasks containing 1 liter of iron-limited HMM media with 1.7 mM sodium citrate. These cultures were grown at 26°C, shaking at 250 rotations per minute (rpm) for 4 days. Cells were removed by centrifugation at 5000 rpm for 30 min. Rotary evaporation was used to concentrate the supernatant to 10 ml. The supernatant was then brought to a 90% methanol concentration and filtered through 125 mm Whatman paper followed by a 0.2 µm filter. The filtered supernatant was diluted 1∶1 with ethyl acetate. Column chromatography was performed using a column of silica resin (SiliaFlash F60 40–63 µm, 230–400 mesh, SiliCycle, Quebec City, Canada). The column was washed with two column volumes of 10∶9∶1 solution ethyl acetate/methanol/water, and eluted in 1 liter of 9∶1 methanol/water. The eluted achromobactin was rotary evaporated to a final volume of 3 ml. Each strain was purified and analyzed using mass spectrometry for three biological replicates.

### Mass Spectrometry

Siderophore analysis was accomplished by liquid chromatography- electro spray ionization-time of flight- mass spectrometry (LC-ESI TOF MS) analysis using an Agilent 1260 HPLC equipped with a binary pump, thermostated autosampler, heated column compartment and diode array detector in line with a MicroToF-QII MSD (Bruker Daltonics, Billerica, MA) equipped with an ESI source operating in negative ionization mode monitoring from *m/z* 50–2500. The crude media preparation from above was separated using a Poroshell 120 EC-C18 HPLC column (3.0×100 mm, 2.7 µm, Agilent Technologies, Santa Clara CA) held at 30°C with the following program using a flow rate of 0.5 ml min^−1^, where bottle A was 10 mM *N,N*-dimethylhexylamine, 10 mM ammonium acetate, pH 7.1 and bottle B was 75% methanol/25% water. The column was pre-equilibrated in 100% A for 2 min prior to injection. The mobile phase was held at 100% A for 5 min and then changed to 100% B over the following 15 min using a linear gradient, at which time the mobile phase was held at 100% B for 5 additional min followed by recycling the mobile phase to 100% A over 2 min and the column was held at 100% A for 2 min to equilibrate the column prior to the next injection. The MSD had the following settings: Capillary, 3000 V; End plate offset, −500 V; Nebulizer gas, 3.0 bar; Drying gas, 10 L/min; Drying gas temperature, 200°C; Funnel 1 RF, 300 Vpp; Funnel 2 RF, 300 Vpp; ISCID energy, 0.0 eV; Hexapole RF, 300 Vpp; Quadrapole Ion energy, 5 eV; Low mass filter, 300 *m/z*; Collision cell RF, 300 Vpp; Collison energy, 8.0 eV, Transfer time, 100.0 µsec; Prepulse storage, 10.0 µsec. The MSD was calibrated using ESI tune mix-low solution (Agilent Technologies) prior to running a set of samples and each run contained an internal standard derived from an automatic injection of 20 µl sodium acetate (0.4 mg/ml). Data was processed using DataAnalysis 4.0 software (Bruker Daltonics, Billerica, MA).

*N,N*-dimethylhexylamine, sodium acetate, ammonium acetate were purchased from Sigma-Aldrich and used without purification. All solvents used during LC-MS analysis were of LC-MS grade.

### Plant Pathogenicity Assays

In order to test the ability of B728a *ΔacsS* to multiply in planta and cause disease, vacuum infiltration of 2-week-old Blue Lake 274 (Burpee Seeds, Warminster, PA) bean plants (*Phaseolus vulgaris* L.) and 4-week-old *Nicotiana benthamiana* was performed. B728a was used as a positive control for this experiment and B728a *ΔgacS* served as our negative control. The bacterial strains were cultured overnight from an isolated colony in 5 ml of LB liquid media at 26°C with shaking at 200 rpm. The overnight cultures were used to inoculate flasks of 100 ml LB liquid, which were grown at 26°C with shaking at 200 rpm to an OD_600_ of 0.6. Cultures were pelleted at 5000 rpm for 10 min at room temperature. Cell pellets were washed in sterile distilled water. After washing cell pellets were resuspended to an OD_600_ of 0.3, which is equivalent to 5×10^8^ CFU ml^−1^. Bacterial suspensions of 5×10^6^ CFU ml^−1^ were made in sterile distilled water with 1% Silwet L-77 (Vac-In-Stuff) surfactant (Lehle Seeds, Round Rock, TX). Plants were suspended in the inoculums and a vacuum was established. The vacuum was held at 20 in Hg for 1 min and slowly released. Plants were rinsed with distilled water and allowed to air dry. The plants were maintained at 25°C in a growth chamber for 72 h. Each strain was analyzed on no less than three plants of each species, and the experiment was independently replicated three times.

To evaluate the ability of the bacterial strains to replicate in planta population analyses were performed for B728a, B728a *ΔacsS*, and B728a *ΔgacS* on Day 0, Day 2, and Day 4 after vacuum infiltration. A trifoliate leaf was selected and detached from each infiltrated plant and infiltrated tissue was removed using the bottom of a sterile 2 ml screw-cap microcentrifuge tube (BioPlas Inc., San Francisco, CA). Twenty leaf discs were removed per leaf and rinsed in sterile distilled water. Leaf discs were ground using a mortar and pestle with Silwet Phosphate Magnesium Buffer (SPM; 0.7% K_2_HPO_4_, 0.4% KH_2_PO_4_, 0.025% MgSO_4_·7 H_2_0, 0.004% Silwet L-77). Serial dilutions were made and plated on KB agar plates followed by incubation at 26°C for 48 h. Colonies were counted and populations were calculated.

## Results and Discussion

### The achromobactin gene cluster is present in a genomic region that produces multiple secondary metabolite products

Analysis of the 6.1 Mb B728a genome revealed a peptide synthetase rich region of over 186 kb, including the syringomycin/syringopeptin (*syr/syp*) toxin clusters, the achromobactin siderophore cluster (*Psyr_2580*-*Psyr_2595*), and an eight module NRPS (*Psyr_2576–2577*) predicted to encode the lipopeptide syringafactin [33]. By examining sequence similarities to other sequenced *P. syringae* species at both the nucleotide and protein level, it is evident that the genes within this region were likely inherited from different microbial ancestors. The syringafactin cluster (*Psyr_2576–2577*) has >80% similarity, at the nucleotide level, to a group of genes in the DC3000 genome. However, the 14-gene cluster that is predicted to encode the citrate siderophore achromobactin is absent from the DC3000 genome, but has >80% sequence similarity to a gene cluster in *P. syringae* pv. *phaseolicola* strain 1448a. Additionally, the *syr/syp* toxin clusters are not found in strains DC3000 and 1448a. Thus, this region of the B728a genome seems to have independently inherited three separate peptide gene clusters. Interestingly, this region accounts for over 3% of the B728a genome. Given the pattern of this genomic region with apparent blocks of genes that were independently inherited, it is likely that the regulatory genes located within these peptide clusters are associated with the regulation of that specific peptide. Accordingly, we hypothesized that the ECF σ^70^ factor encoded on gene *Psyr_*2580, is directly involved in the regulation of the achromobactin gene cluster in response to iron-limited conditions. This gene has been designated as the achromobactin sigma factor, *acsS*.

### The *acsS* sigma factor gene is specific to pseudomonad achromobactin clusters

**Figure 1:**
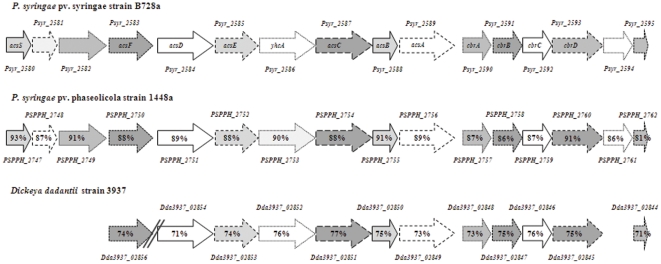
Sequence homology of the achromobactin gene clusters in *P. syringae* pv. syringae B728a, *P. syringae* pv. phaseolicola 1448a, and *Dickeya dadantii* strain 3937. Sequence homology is shown as a percentage and is in reference to the B728a genome with homologous genes being shown in the same shade and line pattern. Parallel lines reflect a gap in the genome, wherein the genome shown has nucleotides and/or genes that are not found in that location in strain B728a.

The 19.9 kb achromobactin gene cluster (*Psyr_2580*-*Psyr_2595*) of B728a has a homologous gene cluster in 1448a with 88% similarity at the nucleotide level. This homologous gene cluster in 1448a includes a gene, *PSPPH_2747*, with 93% similarity at the nucleotide level to the ECF sigma factor gene *acsS* (Fig. 1). Furthermore, the achromobactin clusters in B728a (*Psyr_2580*-*Psyr_2595*) and 1448a (*PSPPH_2747-PSPPH_2762*) maintain sequence homology above 80% at the nucleotide level for each of the 16 genes in the cluster, as well as, gene order and directionality [34].

The achromobactin biosynthesis cluster from *Dickeya dadantii* strain 3937 is 20.6 kb in length and is 86% similar at the nucleotide level to the cluster found in *Dickeya zeae* strain Ech1591. When the entire achromobactin biosynthesis and secretion regions are compared, very little sequence similarity is seen between these *Dickeya* strains and the fluorescent pseudomonads (Fig. 1). However, when analyzed for individual gene homology, 12 of the 16 genes in the B728a achromobactin cluster have over 70% sequence similarity at the nucleotide level to the *Dickeya* strains. These genes include those necessary for achromobactin biosynthesis, *acsD*, *acsE*, *acsC*, and *acsA* [13], as well as the ABC transporter system encoded by genes *cbrABCD*. The sequence similarity of these gene clusters, as well as previous research indicating that the achromobactin synthesized by B728a can be recognized and utilized by *Dickeya dadantii* strain 3937 [8], supports that these genes are homologs to the achromobactin biosynthesis and secretion genes identified in *Dickeya*. Interestingly, the *Dickeya* strains do not contain homologs to the ECF sigma factor *acsS;* therefore, it is likely that achromobactin biosynthesis is regulated differently in B728a than in the *Dickeya* strains.

In order to test the hypothesis that the ECF σ^70^ factor encoded within the achromobactin gene cluster is directly involved in the regulation of achromobactin biosynthesis and secretion, a deletion mutant, B728a Δ*acsS*, was created using a modified Red Recombinase strategy.

### The AcsS sigma factor does not play a significant role in plant disease

To investigate the role of the AcsS sigma factor in planta, 2-week-old bean plants (*Phaseolus vulgaris* L.) and 4-week-old *Nicotiana benthamiana* were vacuum infiltrated with bacterial cultures. The infiltrated plants were visually inspected at day 0, day 2, and day 4 for disease symptoms and in planta colonization was analyzed on each of these days by population counts. The B728a *ΔacsS* infiltrated strains displayed disease symptoms equivalent to those seen with the wild type strain, B728a. Those plants inoculated with the negative control strain, B728a *ΔgacS*, did not exhibit any disease symptoms. The B728a *ΔacsS* and B728a strains had equivalent populations in the leaf tissue as determined by CFU per area of infected leaf tissue. Based on these analyses, it appears that AcsS is not necessary for the establishment and development of plant disease. Similarly, achromobactin is not required for the development of halo blight disease symptoms by *P. syringae* pv. phaseolicola strain 1448a [34]. Since there is no apparent disease phenotype, expression analysis was used to further pursue the characterization of AcsS.

### The *acsS* gene is responsive to low iron environmental conditions

Prior to intensive expression analysis studies, the iron responsiveness of *acsS* was verified using quantitative real time reverse transcription PCR (qRT-PCR). Expression analysis by qRT-PCR showed that *acsS* is upregulated in low iron conditions and that expression was downregulated in both the 10 µM and 100 µM iron added conditions. Expression of *acsS* was approximately 10 fold higher in the HMM with limited iron than in either of the conditions containing larger concentrations of iron. The increased expression of *acsS* in low iron conditions supported the postulation that AcsS is the sigma factor responsible for the regulation of the achromobactin gene cluster.

### RNA-seq analysis provides a transcriptome-wide view of the AcsS regulon

Initially, high-throughput sequencing technologies for RNA-seq, such as Roche 454 GS FLX, Illumina Genome Analyzer, and Life Technologies ABI SOLiD were primarily limited to eukaryotic organisms, due to the ease of enriching mRNA from these organisms [35], [36], [39]. Less than 5% of the cellular RNA is mRNA [39]; therefore, performing transcriptome analysis with total RNA resulted in an abundance of sequencing results for the ribosomal RNA and tRNA with very few reads mapping to mRNA targets. The absence of a 3′-end poly(A) tail in prokaryotes presents a challenge for enriching mRNA in these systems; however, recent technological advancements allow for the elimination of a large percentage of the ribosomal RNA in many Gram positive and Gram negative prokaryotic systems [39]. The Ambion MicrobExpress™ system removes 16 S and 23 S ribosomal RNA from prokaryotic total RNA samples by hybridization to magnetic beads and was utilized in this study [27], [36], [28].

In an RNA-seq study, after gathering total RNA and removing ribosomal RNA, the enriched mRNA is converted into a cDNA. Traditionally double stranded cDNA has been used for these analyses; however, several recent studies have utilized single stranded cDNA in order to capture transcript directionality and isolate transcript signal strength by DNA strand [27], [28]. In this study, the impact of the *acsS* mutation on the B728a transcriptome was analyzed using RNA-seq analysis of single stranded cDNA libraries on an Illumina Genome Analyzer platform. In order to investigate the regulatory role of the AcsS sigma factor on iron responsive genes, including the achromobactin cluster, B728a and B728a *ΔacsS* were grown in iron-limited HMM media and total RNA was extracted. Due to the nature of RNA-seq analysis, in which contaminating DNA could lead to vastly misleading results, and the rapid degradation of prokaryotic mRNA, total RNA quality was strictly monitored. Pooling of biological samples for hybridization based transcriptome analyses has been shown to increase the efficiency and cost-effectiveness of these analyses while continuing to provide equivalent statistical power [30]. Similarly, RNA-seq using the Illumina sequencing platform was analyzed for technical reproducibility and the researchers concluded that a single mRNA sample run once in a single flow cell would provide sufficient data in many experimental design circumstances [37]. Taking these analyses into consideration [37], [38], this study used a pooled sample of three biological replicates for each strain. The samples were pooled as enriched mRNA, prior to first strand cDNA synthesis.

### Sequencing libraries were constructed from the single stranded cDNA samples and subjected to sequencing on the Illumina GAIIX platform

A total of 21,295,605 reads were acquired for the B728a *ΔacsS* sample. After trimming, there were 20,936,474 million reads. The B728a sample resulted in a total of 9,813,229 reads. After trimming, there were 9,651,997 million reads. The variability between the total number of reads for the wild type and mutant strains is likely due to the challenges associated with accurately quantifying single stranded DNA. Alignments to the B728a genome were generated using CLC Genomics Workbench (V4.5, CLC Bio.). The B728a sample resulted in a total of 5,987,236 mapped reads (2,285,461 uniquely and 3,701,775 non-specific) and a total of 3,664,761 unmapped reads. The B728a *ΔacsS* sample resulted in a total of 13,220,583 mapped reads (4,480,571 unique and 8,740,012 non-specific) and a total of 7,715,891 unmapped reads. Many of the unmapped reads were further analyzed by BLAST analysis and associated with poorly annotated regions of the B728a genome, such as bacteriophage elements. Reads that mapped uniquely to the B728a genome were used to calculate the normalized gene expression as Reads per Kilobase per Million Mapped Reads (RPKM) [31].

### 287 genes were identified that were differentially expressed between B728a and B728a *ΔacsS*

The differential gene expression of the pooled samples from each condition was analyzed using the R sequence package DEGseq, which models the RNA-seq data as a random sampling process and utilizes the assumption that a binomial distribution can be used for the number of reads resulting from a gene [32]. Using this statistical method the data from the two samples was normalized despite the variation in the number of uniquely mapped reads. The robustness of this statistical approach is supported by the fact that known B728a reference genes, such as the *recA* housekeeping gene, are not differentially expressed between the wild type and mutant samples. Using a stringent p-value of less than 0.001, 287 genes were identified that were differentially expressed between B728a and B728a *ΔacsS* (Table S1). Ribosomal RNA genes were retracted from this dataset.

### The sigma factor AcsS regulates achromobactin biosynthesis and transport

The Illumina RNA-seq analysis of B728a and B728a *ΔacsS* in limited iron conditions provided secondary confirmation of the effective deletion of *acsS*, as the wild type strain had 923 fold higher expression of this gene than the B728a *ΔacsS* strain (Table 3). Transcriptome analysis lends support to the hypothesis that the sigma factor AcsS is a regulator of achromobactin biosynthesis and transport. As seen in Table 3, 12 of the 15 genes in the achromobactin gene cluster had decreased gene expression in the B728a *ΔacsS* strain. Additionally, the fold change associated with the achromobactin biosynthesis genes was the most substantial, with the achromobactin transporter YhcA having a 41.1 fold decrease in gene expression in B728a *ΔacsS* as compared to the wild type. The achromobactin biosynthesis genes also showed substantial decreases in gene expression in the *acsS* mutant strain with decreases in fold change ranging from 14.9 fold (*acsA*) to 39.6 fold (*acsD*). The TonB-dependent siderophore receptor encoded on *Psyr_2582*, had an 18.4 fold lower expression in the *acsS* mutant. Transcriptome analysis confirms that the deletion of the *acsS* sigma factor gene disrupts the gene expression of the gene cluster responsible for the biosynthesis and transport of the siderophore achromobactin.

**Table 3:** RNA-seq analysis of the achromobactin gene cluster.

Gene	Locus Tag	Operon^a^	Functional Category	Gene Product	Fold Change^b^	P-value
*acsS*	*Psyr_2580*	508	Transcription	sigma 70 DNA-dependent RNA polymerase subunits	923.1559	1.20E-24
	*Psyr_2581*	508		FecR-like		
	*Psyr_2582*		Inorganic ion transport and metabolism	Ton-B dependent siderophore receptor	18.368	4.04E-75
*acsF*	*Psyr_2583*		Amino acid transport and metabolism	Diaminobutyrate-2-oxoglutarate aminotransferase	25.581	1.92E-281
*acsD*	*Psyr_2584*	509	Secondary metabolites biosynthesis, transport and catabolism	IucA/IucC, Achromobactin synthesis	40.0539	1.12E-188
*acsE*	*Psyr_2585*	509	Amino acid transport and metabolism	Orn/DAP/Arg decarboxylase 2:Orn/DAP/Arg decarboxylase 2	32.2687	3.74E-153
*yhcA*	*Psyr_2586*	509	Carbohydrate transport and metabolism	EmrB/QacA family drug resistance transporter	41.1603	1.62E-60
*acsC*	*Psyr_2587*	510	Secondary metabolites biosynthesis, transport and catabolism	IucA/IucC	30.1028	3.31E-87
*acsB*	*Psyr_2588*	510	Carbohydrate transport and metabolism	HpcH/HpaI aldolase	39.6891	2.61E-64
*acsA*	*Psyr_2589*	510	Secondary metabolites biosynthesis, transport and catabolism	IucA/IucC	14.9494	4.98E-80
*cbrA*	*Psyr_2590*	510	Inorganic ion transport and metabolism	Periplasmic binding protein	4.5983	1.24E-16
*cbrB*	*Psyr_2591*	510	Inorganic ion transport and metabolism	Transport system permease protein	3.2514	3.30E-05
*cbrC*	*Psyr_2592*	510	Secondary metabolites biosynthesis, transport and catabolism	Transport system permease protein		
*cbrD*	*Psyr_2593*	511	Secondary metabolites biosynthesis, transport and catabolism	ABC transporter		
	*Psyr_2594*	511		Hypothetical	2.241	0.000336643
	*Psyr_2595*	511	Coenzyme transport and metabolism	Menaquinone biosynthesis	2.6833	1.52E-05

a Operon predictions were made using the database of prokaryotic operons (http://csbl1.bmb.uga.edu).

b Fold change is reflected as *P. syringae* pv. syringae B728a in comparison to the *ΔacsS* deletion mutant; therefore, a positive fold change reflects a decreased level of gene expression in *P. syringae* pv. syringae B728a *ΔacsS*.

### Regulation of achromobactin biosynthesis by AcsS is confirmed by mass spectrometry

**Figure 2:**
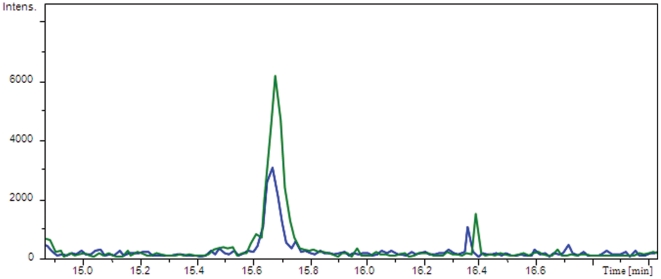
LC-ESI TOF MS analysis of achromobactin isolated from the *acsS* mutant. Samples were purified from culture supernatant of the following strains grown in HMM+1.7 mM sodium citrate for four days: *P. syringae* pv. syringae B728a (green line) and *P. syringae* pv. syringae B728a *ΔacsS* (blue line). This sample analysis is representative of the results from three separate purifications and siderophore analyses for each sample.

Although the RNA-seq analysis confirms that AcsS regulates the achromobactin gene cluster, further analysis was necessary to confirm an impact on the production of the molecular product. By slightly modifying the purification procedure of Berti and Thomas [13] quantifiable achromobactin samples were obtained. Siderophore analysis was accomplished by liquid chromatography-electro spray ionization-time of flight-mass spectrometry (LC-ESI-TOF-MS). Based upon the ESI-MS study performed by Berti and Thomas [13], the mass ions of achromobactin are [M-H] ^−1^ 590.14, [M-2H] ^−2^ 294.57. Achromobactin was purified from the supernatant of one-liter cultures of B728a and B728a *ΔacsS* using the modified purification procedure [13]. LC-ESI TOF MS analysis revealed that B728a *ΔacsS* produces approximately half as much achromobactin as the wild type B728a (Fig. 2). This data confirms that AcsS regulates the production of achromobactin; however, since achromobactin levels were only severely reduced but not eliminated, it implies that there may be other regulators of the citrate siderophore. Given the interdependence of siderophore systems in *Dickeya dadantii* strain 3937, it is conceivable B728a's siderophore systems are also interconnected. Therefore, the pyoverdine regulating sigma factor, PvdS, would be a logical regulatory factor to evaluate.

### Iron responsive genes are regulated by AcsS

After confirming that AcsS is responsive to low iron conditions and regulates the gene expression of the achromobactin siderophore biosynthesis cluster, we hypothesized that other iron responsive genes may also be regulated by the AcsS sigma factor. Amongst the 287 genes differentially expressed between B728a and the *acsS* deletion strain, 31 genes with known associations with iron uptake and metabolism were identified (Table S1). When combined with the achromobactin biosynthesis and transport genes shown to be regulated by AcsS, a total of 43 of the 287 differentially expressed genes identified in the RNA-seq analysis are predicted or known to be associated with iron. This includes an RNA polymerase sigma factor gene, *Psyr_4731*, located in an operon with a gene predicted to encode an iron sensor molecule. Although the fold change for this operon is approximately two, given the regulatory role of sigma factors this could have substantial impact on downstream gene targets. Since the sigma factor gene *Psyr_4731* is expressed at a higher level in the wild type B728a than in the *acsS* deletion mutant, it is likely that some of the differentially expressed genes identified in this study may also be downstream of the sigma factor *Psyr_4731*. Another regulatory gene, *Psyr_2141*, was also differentially expressed in this study and had a 1.5 fold reduction in the *acsS* deletion mutant as compared to the wild type. *Psyr_2141*, which is orthologous to genes in DC3000 and 1448a, encodes a protein with a conserved domain containing sequence homology to proteins in the Fur (ferric uptake regulator) family [36]. Thus, AcsS plays a broad-spectrum role in the regulation of iron and is not limited to the biosynthesis and secretion of achromobactin.

Differential gene expression of several iron associated receptors and secretion genes provides evidence that in low iron conditions a complex network of highly regulated uptake and secretion systems is necessary to maintain cellular functioning (Table 2). Out of the 19 TonB- dependent receptors located in the B728a genome, four had decreased expression in *acsS* deletion mutant, *Psyr_2582*, *Psyr_1962*, *Psyr_3243*, and *Psyr_3345* [40]. Amongst these, *Psyr_2582* showed the largest fold change, an 18.4 fold decrease in the B728a *ΔacsA* strain, not surprising given its genomic location within the achromobactin cluster. However, *Psyr_1962* is encoded within the gene cluster associated with the biosynthesis and secretion of pyoverdine, and has homologous genes within DC3000 and 1448a [40]. This implies that the regulatory networks of iron responsive genes may be highly intertwined. The two remaining TonB receptors are located outside of known iron associated gene clusters and are also conserved amongst B728a, DC3000, 1448a [40]. Thus, three out of the four TonB-dependent receptors regulated by AcsS are conserved amongst B728a, DC3000, and 1448a, despite the fact that DC3000 does not contain a gene homologous to *acsS* [40]. Thus it is likely that these strains have divergent regulatory networks, despite the conserved gene homology.

Interestingly, this study also showed that many genes necessary for biosynthesis and secretion of the siderophore pyoverdine were also impacted by the *acsS* deletion (Table S1). Although the fold change is relatively small, with most genes showing a 1.5 to 2 fold change, 17 genes associated with pyoverdine biosynthesis and transport had differential gene expression between the *acsS* mutant and the wild type. Moreover, this includes the sigma factor associated with the regulation of pyoverdine biosynthesis, *pvdS*, which had 1.6 fold lower gene expression in the B728a *ΔacsS* strain than in the wild type strain. Therefore, the deletion of the sigma factor AcsS resulted in a decrease in expression of both achromobactin and pyoverdine. This was a surprising result, as it was hypothesized that pyoverdine production would increase in this mutant strain to compensate for the loss of the siderophore achromobactin. Based on this study it appears that the regulation and biosynthesis of pyoverdine and achromobactin are extensively intertwined and that these separate iron acquisition systems work collaboratively in low iron environments, rather than competitively.

### AcsS regulates an RND-type efflux system

In addition to supporting the hypothesis that AcsS regulates the biosynthesis and transport of achromobactin and other iron responsive genes, a benefit of performing an RNA-seq analysis is that it provides a comprehensive analysis of the transcriptome and identified differentially expressed genes that may have never been predicted or anticipated. In order to minimize the possibility of false positives and identify the most likely genes that are directly or indirectly regulated by AcsS, a stringent p-value (0.001) was selected for data analysis in this study. The Resistance-Nodulation-Cell division (RND-type) efflux system encoded in operon 516, *pseABC* (*Psyr_2620–2622*), was identified as having over a 10 fold decrease in gene expression in the *acsS* mutant; therefore, this membrane-spanning transport system may play a role in the secretion of achromobactin and/or other metabolites regulated by the AcsS sigma factor (Table 4).

**Table 4:** RNA-seq analysis of the RND efflux system encoded by the *pseABC* gene cluster.

Gene	Locus Tag	Operon^a^	Gene Product	Fold Change^b^	P-value
*pseA*	Psyr_2620	516	RND efflux system, outer membrane lipoprotein, NodT	11.9614	1.61E-18
*pseB*	Psyr_2621	516	Secretion protein HlyD	19.3136	2.33E-16
*pseC*	Psyr_2622	516	Acriflavin resistance protein	10.5859	3.75E-16

a Operon predictions were made using the database of prokaryotic operons (http://csbl1.bmb.uga.edu).

b Fold change is reflected as *P. syringae* pv. syringae B728a in comparison to the *ΔacsS* deletion mutant; therefore, a positive fold change reflects a decreased level of gene expression in *P. syringae* pv. syringae B728a *ΔacsS*.

RND-type efflux systems are found in prokaryotes, archaea, and eukaryotes and utilize ion gradients to fuel the transport of antibiotics and other compounds across the inner and outer membranes into the environment [41], [42]. These Type I secretion systems utilize an inner membrane proton antiporter, in which the transfer of hydrogen ions drives the export of substrate molecules from the cytosol directly into the extracellular environment [41]. Much of our knowledge about RND-type efflux systems is based on extensive research on the AcrAB-TolC efflux system found in *Escherichia coli* K-12 and *Salmonella enterica* serovar typhimurium SH5014, with the primary focus being the implications of multidrug (antibiotic) resistance [42], [43], [44]. However, RND-type efflux systems are not limited to the transport of antibiotics. Recent studies have diversified our understanding of these transporters and emphasized their roles in not only antimicrobial resistance, but also in environmental adaptation and pathogen virulence. The rice pathogen, *Xanthamonas oryzae* pv. oryzae, requires an RND-efflux system for the transport of the yellow pigment, xanthomonadin, which plays an important role pathogen protection against UV radiation and photo-oxidative stress on the leaf surface [45]. Xanthomonadin deficient mutants have a severely decreased ability to survive epiphytically compared to wild type strains; therefore, the ability of this pathogen to transport xanthomonadin to the extracellular environment is an important component of the pathogen's ability to adapt to the leaf surface environment [45]. Likewise, *P. syringae* pv. syringae B301D and B728a utilize an RND-type efflux system, PseABC, and an ATP-binding cassette (ABC) transporter for the secretion of the lipopeptide phytotoxins, syringomycin and syringopeptin, which serve as major virulence factors for these strains [44]. In addition to involvement in the transport of pigments and phytotoxins, there is some evidence supporting the involvement of RND-type efflux systems in the transport of the *E. coli* siderophore enterobactin [46]. Thus, based on the over 10 fold decrease in gene expression in the B728a *ΔacsS* strain, it can be postulated that the B728a RND-efflux system, PseABC, is being directly regulated by the AcsS sigma factor and is involved in the transport of achromobactin. However, it also possible that the PseABC RND transporter is involved in the transport of other secondary metabolites regulated by the AcsS sigma factor, such as the antimetabolite toxin, mangotoxin.

### AcsS regulates the antimetabolite toxin, mangotoxin

Mangotoxin is a non-host specific antimetabolite toxin that inhibits ornithine acetyl transferase, thereby disrupting the ornithine and arginine biosynthetic pathways [47]. This toxin was first identified as a product of *P. syringae* pv. syringae UMAF0158 (*P.s.s.* UMAF0158), a pathogen that causes apical necrosis of mango [47]. B728a, DC3000, and 1448a contain genes with >80% sequence similarity to the mangotoxin nonribosomal peptide synthetase gene (*mgoA*), as well as neighboring genes that are hypothesized to be involved in the biosynthesis of the antimetabolite toxin [47]. In B728a the gene homologous to *mgoA* (*Psyr_5011*) is encoded within an operon with three hypothetical genes. Interestingly, all four of the genes in this operon were differentially expressed at a statistically relevant level in the RNA-seq analysis; with each having at least 3.9 fold lower expression in the AcsS mutant than in the wild-type strain (Table 5). In the mango pathogen, *P.s.s.* UMAF0158, mangotoxin serves as a virulence factor, wherein mangotoxin deficient strains show a definitive delay in symptom initiation and progression [47], [48]. Mangotoxin deficient strains showed no statistically relevant difference from the wild type strain in their ability to survive epiphytically [48]. However, when co-inoculated on the leaf surface, the mangotoxin deficient strain was less efficient at surviving in the epiphytic environment [48]. Thus, it is hypothesized that mangotoxin plays a role in epiphytic fitness and competitiveness [48]. Mangotoxin biosynthesis by B728a has not been investigated; however, based on this study the genes predicted to be involved in this process (*Psyr_5009*-*Psyr_5012*) are being expressed in limited iron conditions. This study supports that the biosynthesis of this mangotoxin-like product is positively regulated by the sigma factor AcsS. Further studies could elucidate if this product contributes to the survival of B728a on the leaf surface by providing a competitive advantage, similarly to the mangotoxin produced by *P.s.s.* UMAF0158.

**Table 5:** RNA-seq analysis of the mangotoxin gene cluster.

Gene	Locus Tag	Operon^a^	Gene Product	Fold Change^b^	P-value
	*Psyr_5009*	983	Hypothetical protein, mangotoxin biosynthesis	4.7447	4.39E-19
	*Psyr_5010*	983	Hypotherical protein, mangotoxin biosynthesis	4.0186	3.36E-10
*mgoA*	*Psyr_5011*	983	Mangotoxin biosynthesis, amino acid adenylation/thioester reductase	5.2527	2.72E-13
	*Psyr_5012*	983	Hypothetical protein, mangotoxin biosynthesis	3.9592	8.07E-07

a Operon predictions were made using the database of prokaryotic operons (http://csbl1.bmb.uga.edu).

b Fold change is reflected as *P. syringae* pv. syringae B728a in comparison to the *ΔacsS* deletion mutant; therefore, a positive fold change reflects a decreased level of gene expression in *P. syringae* pv. syringae B728a *ΔacS*.

### Synthesis of the exopolysaccharide Psl is positively regulated by AcsS

Although the roles of the B728a exopolysaccharides (EPS) alginate and levan, have been extensively studied, recently a putative novel EPS has been identified in this strain, called Psl [21]. This EPS was first identified in *P. aeruginosa* PAO1 as a galactose and mannose rich EPS that serves as an essential component of biofilm formation, which is an important component of pathogenicity for this organism [49], [50], [51]. It is also known that in *P. aeruginosa* PAO1 environmental iron serves as a signal for biofilm formation and iron uptake is required for such formation to occur [52]. Unfortunately, the role of Psl within the life cycle of B728a has not been determined. However, in this study 6 out of 11 of the genes in the predicted *psl* operons (*Psyr_3301* to *Psyr_3311*) were expressed at approximately 2 fold higher levels in the wild type than in the *acsS* deletion strain (Table S1). Moreover, the Psl biosynthesis clusters are located in three operons and genes from each of these operons are differentially expressed in the RNA-seq analysis. Similarly to what is seen in *P. aeruginosa* PAO1, siderophores and other iron acquisition methods may play an important role in B728a's ability to synthesize EPS and biofilms. Therefore, the biosynthesis of these EPS systems, such as Psl, may be regulated in conjunction with iron acquisition systems. It is not known if Psl is regulated directly by the AcsS sigma factor or if this differential expression is merely a secondary effect of the bacteria's decreased efficiency in iron acquisition. Biofilm formation is a complicated process and involves numerous cellular functions, with iron sensing and acquisition being only a small piece of this process. However, numerous functions known to be involved in biofilm formation were impacted by the *acsS* gene mutation. Another aspect of biofilm formation identified in this study as being impacted by the deletion of the AcsS sigma factor gene is cellular motility.

### Cell motility genes are regulated by AcsS

*P. aeruginoasa* PAO1 cells that are exposed to extreme iron limitation by the addition of lactoferrin, an iron chelator, are incapable of forming mature biofilms [52]. Instead these bacteria convert from a planktonic to a sessile state and display persistent twitching motility; however, complex biofilm structure and EPS production does not occur [52]. In *P. aeruginosa* PA14 flagellar-mediated motility, as well as, twitching motility via Type IV pili are both necessary for mature biofilm formation [53]. Mutants deficient in Type IV pili were only capable of forming a thin layer of cells on the attachment surface and could not form mature biofilm structures [53]. In this study two genes predicted to be associated with Type IV pilus formation were identified that were expressed at higher levels in wild type B728a than in the *acsS* mutant, *Psyr_0799* and *Psyr_1661*. Non-motile strains of *P. aeruginosa* PA14 with mutations in the flagella hook-associated gene, *flgK*, were not capable of making surface attachment and were thus incapable of forming biofilms [53]. The RNA-seq analysis in this study revealed 19 flagella genes with lower gene expression in the *acsS* mutant than in the wild type strain (Table S1). The proteins encoded by these flagella genes consist of major conserved structural elements of the flagella including: the filament (*fliC/Psyr_3466*), the hook-filament junction (*flgK*/*Psyr_3471*, *flgL*/*Psyr_3470*), the hook (*flgE*/*Psyr_*3478), the rod (*flgC*/*Psyr_3480*, *flgG*/*Psyr_3475*, *flgF*/*Psyr_3476*), the MS ring (*fliF*/*Psyr_3457*), the C ring (*fliG*/*Psyr_3456*), and the hook-capping protein (*flgD*/*Psyr_3479*) [54]. Noticeably the *fleS/fleR* genes encoding a two-component regulatory system involved in flagella regulation are also differentially expressed with approximately 2 fold lower expression in the *acsS* mutant than in the wild type [55]. In *P. aeruginosa* PAK and *P. aeruginosa* PAO1 the regulatory hierarchy of flagella genes has been extensively studied and the flagella genes *flgBCDE*, *flgFGHIJKL* and *fliK* are all regulated by the FleS/FleR two-component system [55]. Thus, at least 10 of the flagella genes showing differential gene expression in this study are probably being directly regulated by FleR/FleS, which is also being regulated by AcsS. Further studies are necessary to demonstrate the phenotypic effect that a 1.5 to 2 fold decrease in flagella gene expression has on B728a. However, this finding emphasizes the advantage of RNA-seq analyses in identifying gene expression changes that although not astronomical in numerical value can substantially contribute to our biological understanding of the organism.

### AcsS may contribute to epiphytic survival and competitiveness

This study has shown based on RNA-seq analysis that the sigma factor AcsS is involved in the regulation of the siderophores achromobactin and pyoverdine, several iron transporter systems, TonB-siderophore receptors, an RND-efflux system, mangotoxin-like biosynthesis, Psl biosynthesis, Type IV pili, and cell motility. When considered in totality these factors support the hypothesis that achromobactin and AcsS may play an important role in the epiphytic stage of the B728a lifecycle. This coincides with previous studies in which the siderophore achromobactin was shown to be important for the epiphytic survival of *P. syringae* pv. syringae strain 22d/93 [8]. Like DC3000, during the infection process of its life cycle B728a establishes populations in the apoplastic spaces of leaf tissue. Although it has not been reported for B728a, studies have shown that siderophore systems are not necessary for the virulence and disease by DC3000 and 1448a, suggesting that sufficient iron is present in the apoplastic spaces for pathogen survival and function [7], [34]. Thus, it is likely that the available iron in the apoplastic spaces is also sufficient for B728a to survive and cause disease. However, unlike DC3000, B728a is an efficient epiphyte and this study suggests that the siderophore achromobactin may play a significant role in the survival of B728a on the leaf surface. The completion of a transcriptome analysis of the B728a *ΔacsS* strain has provided insightful leads into the complex iron regulatory mechanisms of B728a which may involve an over-lapping regulatory network between the pyoverdine and achromobactin systems. Further studies are needed to delineate these regulatory networks and define the role of siderophores on the leaf surface.

## Supporting Information

Table S1 **RNA-Seq analysis of** ***P. syringae*** **pv. syringae. B728a and** ***P. syringae*** **. pv. syringae B728a** ***ΔacsS*** **.**
(DOCX)
