# Supplementary material for: RNA-seq Analysis Reveals That an ECF σ Factor, AcsS, Regulates Achromobactin Biosynthesis in Pseudomonas syringae pv. syringae B728a
Source: PLoS One. 2012 Apr 18;7(4):e34804. doi: 10.1371/journal.pone.0034804 (PMC3329529; doi:10.1371/journal.pone.0034804)
Supplement: Table S1 — RNA-Seq analysis of P. syringae pv. syringae. B728a and P. syringae . pv. syringae B728a ΔacsS . (DOCX) [file pone.0034804.s001.docx]

| **Table S1.** RNA-Seq analysis of *P. syringae* pv. syringae*.* B728a and *P. syringae.* pv. syringae B728a *ΔacsS*.  **Gene** | **Locus Tag** | **Op^a^** | **Function** | **RPKM *P.s.s.* B728a^b^** | **RPKM *P.s.s.* B728a *ΔacsS*^b^** | **log2 (Fold_change) normalized^d^** | **Fold Change^c^** | **z-score^d^** | **p-value^d^** | **q-value^d^** | **q-value^d^** |
| --- | --- | --- | --- | --- | --- | --- | --- | --- | --- | --- | --- |
| ***acsS*** | *Psyr_2580* | 508 | Sigma 70 RNA polymerase | 136.893 | 0.000 | 9.850 | **923.156** | 10.248 | **1.20E-24** | 1.60E-22 | 1.46E-22 |
| ***yhcA*** | *Psyr_2586* | 509 | EmrB/QacA family drug resistance transporter; Acr | 249.999 | 6.075 | 5.363 | **41.160** | 16.410 | **1.62E-60** | 5.99E-58 | 5.46E-58 |
| ***acsD*** | *Psyr_2584* | 509 | IucA/IucC; Acr biosynthesis | 797.588 | 19.916 | 5.324 | **40.054** | 29.297 | **1.12E-188** | 1.93E-185 | 1.76E-185 |
| ***acsB*** | *Psyr_2588* | 509 | HpcH/HpaI aldolase; Acr | 266.547 | 6.717 | 5.311 | **39.689** | 16.932 | **2.61E-64** | 1.04E-61 | 9.48E-62 |
| ***acsE*** | *Psyr_2585* | 509 | Orn/DAP/Arg decarboxylase 2:Orn/DAP/Arg decarboxylase 2; Acr biosynthesis | 653.997 | 20.271 | 5.012 | **32.269** | 26.362 | **3.74E-153** | 3.23E-150 | 2.94E-150 |
| ***acsC*** | *Psyr_2587* | 510 | IucA/IucC; Acr biosynthesis | 370.861 | 12.322 | 4.912 | **30.103** | 19.795 | **3.31E-87** | 2.14E-84 | 1.95E-84 |
| ***acsF*** | *Psyr_2583* |  | diaminobutyrate--2-oxoglutarate aminotransferase; Acr | 1236.351 | 48.339 | 4.677 | **25.581** | 35.849 | **1.92E-281** | 9.96E-278 | 9.07E-278 |
| ***pseB*** | *Psyr_2621* | 516 | secretion protein HlyD | 67.474 | 3.494 | 4.272 | **19.314** | 8.204 | **2.33E-16** | 1.77E-14 | 1.62E-14 |
|  | *Psyr_2582* |  | Ton-B dependent siderophore receptor; predicted Acr secretion | 340.215 | 18.525 | 4.199 | **18.368** | 18.339 | **4.04E-75** | 1.90E-72 | 1.74E-72 |
| ***acsA*** | *Psyr_2589* | 510 | IucA/IucC; Acr biosynthesis | 379.257 | 25.374 | 3.902 | **14.949** | 18.944 | **4.98E-80** | 2.58E-77 | 2.36E-77 |
| ***pseA*** | *Psyr_2620* | 516 | RND efflux system, outer membrane lipoprotein, NodT | 86.604 | 7.242 | 3.580 | **11.961** | 8.782 | **1.61E-18** | 1.41E-16 | 1.29E-16 |
| ***pseC*** | *Psyr_2622* | 516 | Acriflavin resistance protein | 77.560 | 7.328 | 3.404 | **10.586** | 8.147 | **3.75E-16** | 2.78E-14 | 2.53E-14 |
| ***mgoA*** | *Psyr_5011* | 983 | amino acid adenylation:thioester reductase; predicted mangotoxin | 89.544 | 17.050 | 2.393 | **5.253** | 7.332 | **2.27E-13** | 1.29E-11 | 1.18E-11 |
|  | *Psyr_5009* | 983 | hypothetical; predicted mangotoxin | 143.162 | 30.178 | 2.246 | **4.745** | 8.927 | **4.39E-19** | 4.14E-17 | 3.78E-17 |
| ***cbrA*** | *Psyr_2590* | 510 | periplasmic binding protein | 126.280 | 27.467 | 2.201 | **4.598** | 8.280 | **1.24E-16** | 9.57E-15 | 8.72E-15 |
|  | *Psyr_5010* | 983 | hypothetical; predicted mangotoxin | 81.749 | 20.346 | 2.007 | **4.019** | 6.281 | **3.36E-10** | 1.57E-08 | 1.43E-08 |
|  | *Psyr_5012* | 983 | hypothetical; predicted mangotoxin | 51.150 | 12.922 | 1.985 | **3.959** | 4.934 | **8.07E-07** | 2.30E-05 | 2.10E-05 |
|  | *Psyr_0288* |  | carbonate dehydratase | 48.010 | 13.168 | 1.867 | **3.647** | 4.587 | **4.49E-06** | 0.0001152 | 0.000105 |
| Table S1 Continued.  **Gene** | **Locus Tag** | **Op^a^** | **Function** | **RPKM *P.s.s.* B728a^b^** | **RPKM *P.s.s.* B728a *ΔacsS*^b^** | **log2 (Fold_change) normalized^d^** | **Fold Change^c^** | **z-score^d^** | **p-value^d^** | **q-value^d^** | **q-value^d^** |
| ***cbrB*** | *Psyr_2591* | 510 | transport system permease protein | 44.708 | 13.753 | 1.701 | **3.251** | 4.152 | **3.30E-05** | 0.0007102 | 0.000647 |
|  | *Psyr_2595* | 511 | dimethylmenaquinone methyltransferase | 62.925 | 23.454 | 1.424 | **2.683** | 4.326 | **1.52E-05** | 0.0003594 | 0.000328 |
|  | *Psyr_1136* |  | hypothetical | 61.613 | 23.196 | 1.410 | **2.657** | 4.248 | **2.16E-05** | 0.0004823 | 0.000439 |
|  | *Psyr_3369* | 648 | twin-arginine translocation pathway signal:Tat-translocated enzyme:Dyp-type peroxidase; TAT secretion | 411.180 | 164.543 | 1.322 | **2.499** | 10.449 | **1.48E-25** | 2.08E-23 | 1.90E-23 |
|  | *Psyr_3368* | 648 | hypothetical | 181.767 | 72.831 | 1.320 | **2.496** | 6.939 | **3.95E-12** | 2.05E-10 | 1.87E-10 |
|  | *Psyr_1950* | 383 | ABC transporter | 64.844 | 26.004 | 1.318 | **2.494** | 4.141 | **3.46E-05** | 0.0007407 | 0.000675 |
|  | *Psyr_3632* |  | hypothetical | 58.901 | 24.482 | 1.267 | **2.406** | 3.826 | **0.000130** | 0.0023847 | 0.002173 |
| ***fleR*** | *Psyr_3459* | 665 | helix-turn-helix, Fis-type; regulation of flagella | 62.161 | 25.961 | 1.260 | **2.395** | 3.914 | **9.08E-05** | 0.0016876 | 0.001538 |
|  | *Psyr_4015* |  | hypothetical | 99.538 | 43.932 | 1.180 | **2.266** | 4.704 | **2.55E-06** | 6.85E-05 | 6.24E-05 |
|  | *Psyr_3370* | 648 | hypothetical | 677.237 | 302.118 | 1.165 | **2.242** | 12.146 | **5.99E-34** | 1.15E-31 | 1.05E-31 |
|  | *Psyr_2594* | 511 | hypothetical | 59.100 | 26.377 | 1.164 | **2.241** | 3.585 | **0.000336** | 0.0056705 | 0.005167 |
|  | *Psyr_3023* |  | hypothetical | 862.947 | 396.286 | 1.123 | **2.178** | 13.316 | **1.86E-40** | 4.59E-38 | 4.18E-38 |
|  | *Psyr_3305* | 640 | glycocyl transferase, group 1; predicted EPS Psl | 120.839 | 56.778 | 1.090 | **2.129** | 4.862 | **1.16E-06** | 3.25E-05 | 2.97E-05 |
| ***fdhD*** | *Psyr_4945* | 969 | formate dehyrogenase, subunit FdhD | 75.230 | 35.606 | 1.079 | **2.113** | 3.806 | **0.000141** | 0.0025676 | 0.00234 |
|  | *Psyr_3243* |  | Ton-B dependent siderophore receptor | 78.585 | 37.457 | 1.069 | **2.098** | 3.860 | **0.000113** | 0.0020821 | 0.001897 |
|  | *Psyr_3130* | 607 | Secretion protein HlyD; multidrug efflux | 63.125 | 30.179 | 1.065 | **2.092** | 3.448 | **0.000564** | 0.0088666 | 0.008057 |
|  | *Psyr_1387* |  | ferredoxin--NADP(+) reductase | 89.935 | 43.250 | 1.056 | **2.080** | 4.089 | **4.33E-05** | 0.0008908 | 0.000812 |
|  | *Psyr_3061* | 593 | putative glutathione S-transferase | 65.211 | 31.633 | 1.044 | **2.062** | 3.448 | **0.000564** | 0.0088413 | 0.008057 |
|  | *Psyr_1952* | 383 | hypothetical | 140.216 | 68.836 | 1.027 | **2.037** | 4.988 | **6.10E-07** | 1.78E-05 | 1.62E-05 |
|  | *Psyr_1938* | 382 | response regulator receiver | 71.478 | 35.136 | 1.025 | **2.035** | 3.556 | **0.000376** | 0.0062432 | 0.005689 |
| Table S1 Continued.  **Gene** | **Locus Tag** | **Op^a^** | **Function** | **RPKM *P.s.s.* B728a^b^** | **RPKM *P.s.s.* B728a *ΔacsS*^b^** | **log2 (Fold_change) normalized^d^** | **Fold Change^c^** | **z-score^d^** | **p-value^d^** | **q-value^d^** | **q-value^d^** |
|  | *Psyr_4731* | 927 | RNA polymerase sigma factor | 160.586 | 79.283 | 1.019 | **2.026** | 5.303 | **1.14E-07** | 3.62E-06 | 3.30E-06 |
| ***fleS*** | *Psyr_3460* | 665 | flagellar sensor histidine kinase FleS | 81.380 | 40.279 | 1.015 | **2.021** | 3.764 | **0.000167** | 0.0030222 | 0.002754 |
| ***gidA*** | *Psyr_5132* | 1007 | tRNA uridine 5-carboxymethylaminomethyl modification enzyme GidA | 107.554 | 53.303 | 1.013 | **2.018** | 4.321 | **1.56E-05** | 0.0003669 | 0.000334 |
| ***pslB*** | *Psyr_3302* | 639 | mannose-1-phosphate guanylyltransferase/mannose-6-phosphate isomerase; predicted EPS Psl | 97.601 | 48.507 | 1.009 | **2.012** | 4.102 | **4.09E-05** | 0.0008594 | 0.000783 |
|  | *Psyr_0784* | 157 | CheW-like protein | 146.608 | 73.539 | 0.996 | **1.994** | 4.973 | **6.59E-07** | 1.90E-05 | 1.73E-05 |
|  | *Psyr_4730* | 927 | FecR protein, Fe dicitrate sensor, membrane component | 154.461 | 77.561 | 0.994 | **1.992** | 5.098 | **3.44E-07** | 1.04E-05 | 9.44E-06 |
| ***pvdO*** | *Psyr_1964* | 385 | hypothetical | 539.980 | 272.896 | 0.985 | **1.979** | 9.460 | **3.08E-21** | 3.40E-19 | 3.10E-19 |
|  | *Psyr_2053* | 405 | moxR protein, putative | 74.778 | 38.232 | 0.968 | **1.956** | 3.470 | **0.000521** | 0.0083173 | 0.007579 |
|  | *Psyr_1953* | 383 | hypothetical | 137.429 | 70.415 | 0.965 | **1.952** | 4.691 | **2.71E-06** | 7.26E-05 | 6.61E-05 |
|  | *Psyr_4393* | 853 | secretion | 68.563 | 35.323 | 0.957 | **1.941** | 3.291 | **0.000998** | 0.0147201 | 0.013384 |
|  | *Psyr_4255* |  | FKBP-type peptidyl-prolyl isomerase, N-terminal:peptidylprolyl isomerase, FKBP-type | 536.200 | 276.244 | 0.957 | **1.941** | 9.206 | **3.40E-20** | 3.39E-18 | 3.09E-18 |
|  | *Psyr_2434* |  | carbohydrate kinase, PfkB, fructokinase | 106.368 | 54.857 | 0.956 | **1.939** | 4.094 | **4.25E-05** | 0.0008778 | 0.0008 |
|  | *Psyr_4260* | 829 | hypothetical | 1027.066 | 529.941 | 0.955 | **1.938** | 12.720 | **4.60E-37** | 1.04E-34 | 9.46E-35 |
| ***fleN*** | *Psyr_3438* | 661 | flagellar synthesis regulator, cobyrinic acid a,c-diamide synthase | 138.985 | 71.740 | 0.954 | **1.938** | 4.674 | **2.95E-06** | 7.80E-05 | 7.11E-05 |
|  | *Psyr_3308* | 641 | glycosyl transferase, group 1; predicted EPS Psl | 103.156 | 53.930 | 0.936 | **1.913** | 3.962 | **7.44E-05** | 0.001434 | 0.001307 |
|  | *Psyr_3303* | 640 | polysaccharide export protein; predicted EPS Psl | 78.158 | 40.923 | 0.934 | **1.910** | 3.442 | **0.000577** | 0.0090031 | 0.008186 |
|  | *Psyr_0374* |  | phosphorylase | 247.501 | 129.677 | 0.933 | **1.909** | 6.120 | **9.36E-10** | 4.08E-08 | 3.72E-08 |
|  | *Psyr_3309* | 641 | hypothetical; predicted EPS Psl | 74.782 | 39.209 | 0.932 | **1.908** | 3.361 | **0.000777** | 0.0117231 | 0.010683 |
| Table S1 Continued.  **Gene** | **Locus Tag** | **Op^a^** | **Function** | **RPKM *P.s.s.* B728a^b^** | **RPKM *P.s.s.* B728a *ΔacsS*^b^** | **log2 (Fold_change) normalized^d^** | **Fold Change^c^** | **z-score^d^** | **p-value^d^** | **q-value^d^** | **q-value^d^** |
|  | *Psyr_4376* |  | LuxR response regulator receiver | 201.766 | 106.256 | 0.925 | **1.899** | 5.489 | **4.04E-08** | 1.40E-06 | 1.27E-06 |
|  | *Psyr_4373* | 851 | PAS | 108.344 | 57.257 | 0.920 | **1.893** | 4.004 | **6.24E-05** | 0.0012311 | 0.001122 |
|  | *Psyr_2739* |  | ThiJ/PfpI family protein | 105.284 | 55.669 | 0.920 | **1.892** | 3.944 | **8.02E-05** | 0.0015176 | 0.001383 |
| ***leuC*** | *Psyr_1983* | 390 | isopropylmalate isomerase large subunit | 187.768 | 99.526 | 0.916 | **1.887** | 5.250 | **1.52E-07** | 4.67E-06 | 4.25E-06 |
|  | *Psyr_0458* |  | helicase, C-terminal:Type III restriction enzyme, res subunit:DEAD/DEAH box helicase, N-terminal | 81.832 | 43.423 | 0.914 | **1.885** | 3.461 | **0.000538** | 0.0085497 | 0.007791 |
|  | *Psyr_3345* |  | TonB-dependent siderophore receptor | 211.262 | 112.174 | 0.914 | **1.884** | 5.556 | **2.76E-08** | 9.80E-07 | 8.93E-07 |
|  | *Psyr_1072* |  | extracellular solute-binding protein | 264.573 | 140.547 | 0.913 | **1.883** | 6.214 | **5.16E-10** | 2.37E-08 | 2.16E-08 |
| ***pvdN*** | *Psyr_1966* | 385 | peptidase M19, renal dipeptidase | 534.590 | 284.774 | 0.909 | **1.878** | 8.802 | **1.34E-18** | 1.20E-16 | 1.10E-16 |
| ***gabT*** | *Psyr_0090* |  | 4-aminobutyrate aminotransferase | 124.126 | 66.546 | 0.900 | **1.866** | 4.204 | **2.62E-05** | 0.0005818 | 0.00053 |
|  | *Psyr_0392* |  | hypothetical | 130.452 | 70.201 | 0.894 | **1.859** | 4.288 | **1.81E-05** | 0.0004163 | 0.000379 |
|  | *Psyr_4946* | 969 | oxidoreductase alpha (molybdopterin) subunit; TCA cycle | 197.110 | 106.193 | 0.893 | **1.856** | 5.263 | **1.42E-07** | 4.44E-06 | 4.05E-06 |
| ***pvdN*** | *Psyr_1965* | 385 | twin-arginine translocation pathway signal; secretion | 492.200 | 265.477 | 0.891 | **1.854** | 8.304 | **1.00E-16** | 7.88E-15 | 7.18E-15 |
|  | *Psyr_2462* | 484 | nitrite/sulfite reductase, hemoprotein beta-component, ferrodoxin-like:nitrite and sulphite reductase 4Fe-4S region; sulfur metabolism | 119.100 | 64.378 | 0.888 | **1.850** | 4.072 | **4.66E-05** | 0.0009482 | 0.000864 |
| ***3xrn*** | *Psyr_0579* | 112 | 3'-5' exoribonuclease, VacB and RNase II | 120.796 | 65.790 | 0.877 | **1.836** | 4.058 | **4.94E-05** | 0.0009943 | 0.000905 |
| ***glgA*** | *Psyr_2992* | 580 | trehalose synthesis, glycogen synthase; osmoadaptation | 122.629 | 66.983 | 0.873 | **1.831** | 4.072 | **4.66E-05** | 0.0009509 | 0.000864 |
|  | *Psyr_2799* |  | hypothetical | 420.648 | 231.121 | 0.864 | **1.820** | 7.481 | **7.36E-14** | 4.34E-12 | 3.95E-12 |
|  | *Psyr_1903* | 377 | hypothetical | 82.091 | 45.152 | 0.863 | **1.818** | 3.299 | **0.000969** | 0.014327 | 0.013055 |
|  | *Psyr_0629* |  | aldehyde dehydrogenase | 265.302 | 146.505 | 0.857 | **1.811** | 5.898 | **3.67E-09** | 1.48E-07 | 1.35E-07 |
|  | *Psyr_0785* | 157 | histidine kinase, HAMP region: chemotaxis sensory transducer | 192.842 | 106.699 | 0.854 | **1.808** | 5.014 | **5.32E-07** | 1.58E-05 | 1.44E-05 |
|  | *Psyr_1770* | 360 | enoyl-CoA hydratase/isomerase | 299.818 | 166.641 | 0.848 | **1.799** | 6.212 | **5.23E-10** | 2.38E-08 | 2.17E-08 |
| **Gene**  Table S1 Continued. | **Locus Tag** | **Op^a^** | **Function** | **RPKM *P.s.s.* B728a^b^** | **RPKM *P.s.s.* B728a *ΔacsS*^b^** | **log2 (Fold_change) normalized^d^** | **Fold Change^c^** | **z-score^d^** | **p-value^d^** | **q-value^d^** | **q-value^d^** |
|  | *Psyr_4596* | 897 | hypothetical | 266.882 | 148.478 | 0.846 | **1.798** | 5.853 | **4.84E-09** | 1.92E-07 | 1.74E-07 |
| ***pvdE*** | *Psyr_1963* | 385 | cyclic peptide transporter; pyoverdine ABC transporter, ATP-binding/permease protein | 271.697 | 151.415 | 0.844 | **1.795** | 5.891 | **3.85E-09** | 1.54E-07 | 1.40E-07 |
|  | *Psyr_1477* | 300 | hypothetical | 597.108 | 334.703 | 0.835 | **1.784** | 8.660 | **4.72E-18** | 3.95E-16 | 3.60E-16 |
|  | *Psyr_2634* |  | histidine kinase, HAMP region: chemotaxis sensory transducer | 183.324 | 102.849 | 0.834 | **1.783** | 4.791 | **1.66E-06** | 4.50E-05 | 4.10E-05 |
|  | *Psyr_3372* | 649 | hypothetical | 227.268 | 127.649 | 0.832 | **1.781** | 5.326 | **1.01E-07** | 3.28E-06 | 2.99E-06 |
|  | *Psyr_3060* |  | GAF:ATP-binding region, ATPase-like:histidine kinase A, N-terminal | 132.336 | 74.331 | 0.832 | **1.781** | 4.064 | **4.83E-05** | 0.0009797 | 0.000893 |
|  | *Psyr_0200* |  | hypothetical; predicted signal transduction mechanism | 112.423 | 63.249 | 0.830 | **1.778** | 3.736 | **0.000186** | 0.0033193 | 0.003025 |
| ***pepA*** | *Psyr_1091* |  | leucyl aminopeptidase | 424.332 | 238.739 | 0.830 | **1.778** | 7.260 | **3.88E-13** | 2.19E-11 | 2.00E-11 |
|  | *Psyr_1769* | 360 | Acyl-CoA dehydrogenase, C-terminal:Acyl-CoA dehydrogenase, central region:Acyl-CoA dehydrogenase, N-terminal | 268.511 | 152.724 | 0.814 | **1.758** | 5.680 | **1.34E-08** | 4.91E-07 | 4.47E-07 |
|  | *Psyr_3304* | 640 | lipopolysaccharide biosynthesis; predicted EPS Psl | 104.473 | 59.578 | 0.811 | **1.754** | 3.529 | **0.0004174** | 0.0068101 | 0.006206 |
|  | *Psyr_3024* |  | peptidase M14, carboxypeptidase A | 151.451 | 86.749 | 0.804 | **1.746** | 4.220 | **2.44E-05** | 0.0005434 | 0.000495 |
| ***tssA*** | *Psyr_4966* |  | ImpA, N-terminal; type VI secretion-associated protein | 159.548 | 91.735 | 0.799 | **1.740** | 4.306 | **1.66E-05** | 0.0003899 | 0.000355 |
|  | *Psyr_0391* |  | Poly granule associated | 260.053 | 149.533 | 0.799 | **1.739** | 5.497 | **3.86E-08** | 1.34E-06 | 1.22E-06 |
|  | *Psyr_4150* | 809 | hypothetical | 294.527 | 169.704 | 0.796 | **1.736** | 5.832 | **5.48E-09** | 2.11E-07 | 1.92E-07 |
|  | *Psyr_4676* |  | hypothetical | 165.991 | 95.717 | 0.795 | **1.734** | 4.372 | **1.23E-05** | 0.0002993 | 0.000273 |
|  | *Psyr_4125* |  | hypothetical | 193.730 | 112.437 | 0.785 | **1.723** | 4.676 | **2.93E-06** | 7.79E-05 | 7.10E-05 |
| ***fliF*** | *Psyr_3457* | 664 | flagellar MS-ring protein | 101.617 | 59.198 | 0.780 | **1.717** | 3.366 | **0.000762** | 0.011534 | 0.01051 |
|  | *Psyr_3367* | 648 | Iron permease FTR1 | 268.993 | 157.030 | 0.777 | **1.713** | 5.459 | **4.79E-08** | 1.62E-06 | 1.48E-06 |
|  | *Psyr_1090* | 221 | DNA polymerase III subunit chi | 298.615 | 174.729 | 0.773 | **1.709** | 5.730 | **1.00E-08** | 3.72E-07 | 3.39E-07 |
|  | *Psyr_3373* |  | hypothetical | 1243.845 | 728.437 | 0.772 | **1.708** | 11.686 | **1.51E-31** | 2.70E-29 | 2.46E-29 |
|  | *Psyr_0360* |  | Serine O-acetyltransferase | 229.524 | 134.550 | 0.771 | **1.706** | 5.009 | **5.48E-07** | 1.61E-05 | 1.46E-05 |
|  | *Psyr_4595* | 897 | Bacteriophage Mu tail sheath | 347.656 | 206.809 | 0.750 | **1.681** | 6.018 | **1.77E-09** | 7.53E-08 | 6.86E-08 |
| Table S1 Continued.  **Gene** | **Locus Tag** | **Op^a^** | **Function** | **RPKM *P.s.s.* B728a^b^** | **RPKM *P.s.s.* B728a *ΔacsS*^b^** | **log2 (Fold_change) normalized^d^** | **Fold Change^c^** | **z-score^d^** | **p-value^d^** | **q-value^d^** | **q-value^d^** |
| ***flgJ*** | *Psyr_3472* | 668 | flagellar rod assembly protein/muramidase FlgJ | 204.719 | 121.874 | 0.749 | **1.680** | 4.611 | **4.00E-06** | 0.0001033 | 9.41E-05 |
|  | *Psyr_4221* |  | EAL:GAF | 226.359 | 134.940 | 0.747 | **1.678** | 4.838 | **1.31E-06** | 3.64E-05 | 3.32E-05 |
|  | *Psyr_3592* |  | nitroreductase | 148.563 | 88.583 | 0.746 | **1.677** | 3.918 | **8.94E-05** | 0.0016743 | 0.001526 |
| ***algA*** | *Psyr_1052* |  | mannose-1-phosphate guanylyltransferase/mannose-6-phosphate isomerase; Alginate synthesis | 183.931 | 110.021 | 0.742 | **1.672** | 4.336 | **1.45E-05** | 0.0003486 | 0.000318 |
| ***pyrD2*** | *Psyr_2106* |  | dihydroorotate dehydrogenase 2; pyrimidine biosynthesis | 121.544 | 72.928 | 0.737 | **1.667** | 3.506 | **0.000454** | 0.0073712 | 0.006717 |
|  | *Psyr_2431* |  | zinc-containing alcohol dehydrogenase superfamily protein | 138.220 | 82.968 | 0.737 | **1.666** | 3.736 | **0.000186** | 0.0033295 | 0.003025 |
|  | *Psyr_4254* |  | hypothetical | 379.408 | 227.853 | 0.736 | **1.665** | 6.186 | **6.16E-10** | 2.78E-08 | 2.53E-08 |
|  | *Psyr_3401* | 654 | GCN5-related N-acetyltransferase | 131.593 | 79.166 | 0.733 | **1.663** | 3.632 | **0.000281** | 0.0048038 | 0.004377 |
|  | *Psyr_3129* |  | 3-hydroxyacyl-CoA-acyl carrier protein transferase | 468.396 | 282.511 | 0.730 | **1.658** | 6.823 | **8.91E-12** | 4.58E-10 | 4.17E-10 |
|  | *Psyr_4718* |  | zinc-containing alcohol dehydrogenase superfamily protein; methane metabolism | 278.509 | 168.109 | 0.729 | **1.657** | 5.254 | **1.49E-07** | 4.60E-06 | 4.19E-06 |
|  | *Psyr_3016* | 584 | cobalamin synthesis protein/P47K:cobalamin synthesis protein/P47K | 119.503 | 72.159 | 0.728 | **1.656** | 3.439 | **0.000584** | 0.0090471 | 0.008244 |
|  | *Psyr_2279* |  | hypothetical | 192.871 | 116.611 | 0.726 | **1.654** | 4.359 | **1.31E-05** | 0.0003165 | 0.000287 |
|  | *Psyr_2534* |  | carboxymethylenebutenolidase | 208.025 | 127.173 | 0.710 | **1.636** | 4.440 | **9.00E-06** | 0.0002244 | 0.000204 |
|  | *Psyr_1336* | 272 | tetrahydrodipicolinate succinylase, putative | 164.279 | 100.561 | 0.708 | **1.634** | 3.936 | **8.27E-05** | 0.0015608 | 0.001422 |
|  | *Psyr_0840* |  | pentapeptide repeat-containing protein | 127.808 | 78.490 | 0.704 | **1.629** | 3.452 | **0.000556** | 0.0087812 | 0.008002 |
| ***pvdL*** | *Psyr_1945* |  | peptide synthase | 991.631 | 610.181 | 0.701 | **1.625** | 9.586 | **9.15E-22** | 1.03E-19 | 9.40E-20 |
| ***prfB*** | *Psyr_1310* | 267 | peptide chain release factor 2 | 173.264 | 107.042 | 0.695 | **1.619** | 3.976 | **7.01E-05** | 0.0013621 | 0.001241 |
|  | *Psyr_1303* | 266 | histidine kinase, HAMP region: chemotaxis sensory transducer | 172.065 | 106.725 | 0.689 | **1.612** | 3.933 | **8.38E-05** | 0.0015751 | 0.001435 |
| ***fliO*** | *Psyr_3445* | 663 | flagellar biosynthesis protein, FliO | 123.467 | 76.669 | 0.688 | **1.611** | 3.325 | **0.000884** | 0.0132285 | 0.012054 |
| ***flgG*** | *Psyr_3475* | 668 | flagellar basal body rod protein FlgG | 515.672 | 320.390 | 0.687 | **1.610** | 6.790 | **1.12E-11** | 5.72E-10 | 5.21E-10 |
| TableS1 Continued.  **Gene** | **Locus Tag** | **Op^a^** | **Function** | **RPKM *P.s.s.* B728a^b^** | **RPKM *P.s.s.* B728a *ΔacsS*^b^** | **log2 (Fold_change) normalized^d^** | **Fold Change^c^** | **z-score^d^** | **p-value^d^** | **q-value^d^** | **q-value^d^** |
|  | *Psyr_1608* |  | hypothetical; ice nucleation protein | 754.261 | 471.556 | 0.678 | **1.600** | 8.118 | **4.75E-16** | 3.42E-14 | 3.12E-14 |
| ***ptsN*** | *Psyr_4149* | 809 | PTS IIA-like nitrogen-regulatory protein PtsN; carbohydrate metabolism | 241.374 | 151.279 | 0.674 | **1.596** | 4.569 | **4.89E-06** | 0.0001251 | 0.000114 |
| ***gabD*** | *Psyr_2413* |  | succinate-semialdehyde dehydrogenase (NAD(P)+); GABA metabolism | 172.041 | 108.412 | 0.666 | **1.587** | 3.818 | **0.000134** | 0.0024588 | 0.002241 |
|  | *Psyr_1956* |  | pyoverdine biosynthesis regulatory protein | 5028.420 | 3169.053 | 0.666 | **1.587** | 20.701 | **3.40E-95** | 2.52E-92 | 2.29E-92 |
| ***nadE*** | *Psyr_0594* | 116 | Coenzyme metabolism | 129.906 | 81.943 | 0.665 | **1.586** | 3.311 | **0.000929** | 0.0138154 | 0.012589 |
|  | *Psyr_4631* |  | PrkA serine kinase | 656.797 | 415.526 | 0.661 | **1.581** | 7.405 | **1.31E-13** | 7.63E-12 | 6.95E-12 |
|  | *Psyr_4632* | 907 | hypothetical | 361.224 | 228.584 | 0.660 | **1.581** | 5.488 | **4.06E-08** | 1.39E-06 | 1.27E-06 |
| ***pvdS*** | *Psyr_1943* |  | extracytoplasmic-function sigma-70 factor; regulation of pyoverdine biosynthesis | 432.951 | 274.528 | 0.657 | **1.577** | 5.985 | **2.16E-09** | 9.11E-08 | 8.30E-08 |
|  | *Psyr_4153* | 810 | hypothetical | 1401.972 | 894.250 | 0.649 | **1.568** | 10.653 | **1.69E-26** | 2.50E-24 | 2.28E-24 |
|  | *Psyr_4374* | 851 | TadE family protein; Flp pilus assemble protein TadG | 243.574 | 156.090 | 0.642 | **1.561** | 4.396 | **1.10E-05** | 0.0002722 | 0.000248 |
| ***gapdh*** | *Psyr_1108* |  | glyceraldehyde-3-phosphate dehydrogenase; gluconeogenesis | 2006.597 | 1287.685 | 0.640 | **1.559** | 12.597 | **2.19E-36** | 4.54E-34 | 4.13E-34 |
| ***fliG*** | *Psyr_3456* | 664 | flagellar motor switch protein G | 153.062 | 98.696 | 0.633 | **1.551** | 3.442 | **0.000578** | 0.0089829 | 0.008186 |
| ***lysS*** | *Psyr_1311* | 267 | lysyl-tRNA synthetase | 171.356 | 110.616 | 0.632 | **1.549** | 3.633 | **0.000279** | 0.0047932 | 0.004368 |
|  | *Psyr_1967* |  | twin-arginine translocation pathway signal, TAT secretion | 227.293 | 147.162 | 0.627 | **1.545** | 4.159 | **3.19E-05** | 0.0006903 | 0.000629 |
| ***amrZ*** | *Psyr_3551* |  | Arc-like DNA binding , alginate and motility regulator | 212.481 | 137.632 | 0.627 | **1.544** | 4.018 | **5.88E-05** | 0.0011678 | 0.001064 |
| ***flgH*** | *Psyr_3474* | 668 | flagellar basal body L-ring protein | 205.386 | 133.058 | 0.627 | **1.544** | 3.949 | **7.86E-05** | 0.0015038 | 0.00137 |
|  | *Psyr_0205* | 41 | MotA/TolQ/ExbB proton channel; TonB-system energizer ExbD | 519.624 | 336.647 | 0.626 | **1.544** | 6.282 | **3.35E-10** | 1.58E-08 | 1.43E-08 |
| ***talAB*** | *Psyr_1914* |  | transaldolase B; pentose phosphate pathway | 314.008 | 203.633 | 0.625 | **1.542** | 4.873 | **1.10E-06** | 3.10E-05 | 2.83E-05 |
| ***dat-2*** | *Psyr_1946* |  | diaminobutyrate--2-oxoglutarate aminotransferase; arginine biosynthesis | 1945.280 | 1263.872 | 0.622 | **1.539** | 12.095 | **1.13E-33** | 2.09E-31 | 1.91E-31 |
| ***pvdG*** | *Psyr_1944* |  | thioesterase; pyoverdine synthetase | 1174.875 | 765.664 | 0.618 | **1.535** | 9.335 | **1.01E-20** | 1.05E-18 | 9.53E-19 |
| Table S1 Continued.  **Gene** | **Locus Tag** | **Op^a^** | **Function** | **RPKM *P.s.s.* B728a^b^** | **RPKM *P.s.s.* B728a *ΔacsS*^b^** | **log2 (Fold_change) normalized^d^** | **Fold Change^c^** | **z-score^d^** | **p-value^d^** | **q-value^d^** | **q-value^d^** |
| ***cheA2*** | *Psyr_3434* | 660 | CheW-like protein:ATP-binding region, ATPase-like:Signal transducing histidine kinase, homodimeric:Hpt | 202.524 | 133.673 | 0.600 | **1.515** | 3.770 | **0.000163** | 0.0029569 | 0.002694 |
| ***flgK*** | *Psyr_3471* | 668 | flagellar hook-associated protein FlgK | 240.038 | 158.509 | 0.599 | **1.515** | 4.101 | **4.12E-05** | 0.0008619 | 0.000785 |
|  | *Psyr_1661* |  | Peptidoglycan binding LysM, Tfp pilus assembly protein FimV; Type IV pillus | 182.920 | 120.885 | 0.598 | **1.513** | 3.574 | **0.000352** | 0.0059135 | 0.005389 |
|  | *Psyr_3717* |  | ribonucleotide-diphosphate reductase subunit alpha; pyrimidine salvage | 446.539 | 295.989 | 0.593 | **1.509** | 5.548 | **2.89E-08** | 1.01E-06 | 9.23E-07 |
| ***fliC*** | *Psyr_3466* |  | flagellin | 5379.918 | 3570.565 | 0.592 | **1.507** | 19.268 | **1.00E-82** | 5.76E-80 | 5.25E-80 |
| ***flgD*** | *Psyr_3479* | 669 | flagellar basal body rod modification protein | 860.042 | 570.915 | 0.591 | **1.507** | 7.677 | **1.63E-14** | 1.00E-12 | 9.16E-13 |
| ***fliK*** | *Psyr_3449* |  | flagellar hook-length control protein | 236.377 | 157.205 | 0.589 | **1.504** | 4.007 | **6.16E-05** | 0.0012196 | 0.001111 |
|  | *Psyr_2668* | 521 | helix-turn-helix, Fis-type | 208.031 | 138.611 | 0.586 | **1.501** | 3.743 | **0.000181** | 0.0032496 | 0.002961 |
| ***tkl*** | *Psyr_4792* | 939 | transketolase; involved in glycolysis | 255.799 | 171.155 | 0.580 | **1.495** | 4.112 | **3.91E-05** | 0.0008288 | 0.000755 |
| ***flgE*** | *Psyr_3478* | 669 | flagellar hook protein FlgE | 575.465 | 385.597 | 0.578 | **1.493** | 6.150 | **7.76E-10** | 3.41E-08 | 3.11E-08 |
|  | *Psyr_3614* | 699 | methionine sulfoxide reductase B | 181.362 | 121.673 | 0.576 | **1.491** | 3.442 | **0.000577** | 0.0090242 | 0.008186 |
|  | *Psyr_4388* |  | response regulator receiver | 475.685 | 321.468 | 0.566 | **1.480** | 5.483 | **4.17E-08** | 1.42E-06 | 1.30E-06 |
| ***argJ*** | *Psyr_4093* | 799 | bifunctional ornithine acetyltransferase/N-acetylglutamate synthase protein; arginine biosynthesis | 182.517 | 123.396 | 0.565 | **1.479** | 3.393 | **0.000691** | 0.0105262 | 0.009592 |
| ***yfiA*** | *Psyr_4148* | 809 | sigma 54 modulation protein/ribosomal protein S30EA | 555.118 | 375.750 | 0.563 | **1.478** | 5.902 | **3.59E-09** | 1.45E-07 | 1.33E-07 |
| ***flgL*** | *Psyr_3470* | 668 | flagellar hook-associated protein FlgL | 226.890 | 154.081 | 0.559 | **1.473** | 3.744 | **0.000181** | 0.0032517 | 0.002961 |
| ***flgF*** | *Psyr_3476* | 668 | flagellar basal body rod protein FlgF | 266.875 | 181.290 | 0.558 | **1.472** | 4.058 | **4.96E-05** | 0.0009928 | 0.000905 |
|  | *Psyr_2141* |  | ferric uptake regulator family | 543.140 | 370.499 | 0.552 | **1.466** | 5.733 | **9.84E-09** | 3.67E-07 | 3.35E-07 |
|  | *Psyr_4151* | 809 | phosphocarrier HPr protein | 660.746 | 452.730 | 0.546 | **1.460** | 6.258 | **3.90E-10** | 1.81E-08 | 1.65E-08 |
| ***algR3*** | *Psyr_0054* |  | alginate regulatory protein AlgR3 | 1012.150 | 693.995 | 0.545 | **1.459** | 7.734 | **1.04E-14** | 6.77E-13 | 6.17E-13 |
|  | *Psyr_5080* |  | Cyclopropane-fatty-acyl-phospholipid synthase | 233.803 | 161.403 | 0.535 | **1.449** | 3.654 | **0.000257** | 0.0044573 | 0.004062 |
| Table S1 Continued.  **Gene** | **Locus Tag** | **Op^a^** | **Function** | **RPKM *P.s.s.* B728a^b^** | **RPKM *P.s.s.* B728a *ΔacsS*^b^** | **log2 (Fold_change) normalized^d^** | **Fold Change^c^** | **z-score^d^** | **p-value^d^** | **q-value^d^** | **q-value^d^** |
|  | *Psyr_4633* | 907 | SpoVR family protein | 327.953 | 227.742 | 0.526 | **1.440** | 4.266 | **1.99E-05** | 0.0004515 | 0.000411 |
| ***flgC*** | *Psyr_3480* | 669 | flagellar basal body rod protein FlgC | 640.062 | 445.433 | 0.523 | **1.437** | 5.929 | **3.05E-09** | 1.26E-07 | 1.15E-07 |
|  | *Psyr_2140* |  | hypothetical | 635.390 | 443.833 | 0.518 | **1.432** | 5.852 | **4.86E-09** | 1.91E-07 | 1.74E-07 |
|  | *Psyr_0937* |  | mannose-1-phosphate guanylyltransferase/mannose-6-phosphate isomerase | 258.596 | 181.770 | 0.509 | **1.423** | 3.673 | **0.000239** | 0.0042045 | 0.003831 |
|  | *Psyr_0994* |  | metal-dependent phosphohydrolase | 226.794 | 159.854 | 0.505 | **1.419** | 3.415 | **0.000637** | 0.0097881 | 0.008919 |
| ***pvdI*** | *Psyr_1957* | 384 | amino acid adenylation; pyoverdine sidechain peptide synthetase I, epsilon-Lys module | 1146.385 | 809.861 | 0.502 | **1.416** | 7.637 | **2.22E-14** | 1.35E-12 | 1.23E-12 |
|  | *Psyr_0203* | 41 | TonB, C-terminal | 324.653 | 230.969 | 0.491 | **1.406** | 3.987 | **6.70E-05** | 0.001311 | 0.001195 |
|  | *Psyr_1476* |  | hypothetical | 372.183 | 265.235 | 0.489 | **1.403** | 4.249 | **2.14E-05** | 0.0004816 | 0.000439 |
|  | *Psyr_3654* | 707 | hypothetical | 555.460 | 398.774 | 0.478 | **1.393** | 5.088 | **3.61E-07** | 1.08E-05 | 9.87E-06 |
|  | *Psyr_1872* |  | hypothetical | 754.742 | 544.931 | 0.470 | **1.385** | 5.839 | **5.26E-09** | 2.05E-07 | 1.87E-07 |
| ***clpA*** | *Psyr_3183* | 617 | putative AAA ATPase | 456.630 | 330.499 | 0.467 | **1.382** | 4.509 | **6.51E-06** | 0.0001639 | 0.000149 |
| ***fliS*** | *Psyr_3463* | 666 | flagellar protein FliS | 276.229 | 200.542 | 0.462 | **1.378** | 3.476 | **0.000508** | 0.008145 | 0.007422 |
| ***leuA*** | *Psyr_1257* |  | 2-isopropylmalate synthase; leucine biosynthesis | 299.360 | 217.458 | 0.461 | **1.377** | 3.613 | **0.000302** | 0.0051325 | 0.004677 |
| ***pvdJ*** | *Psyr_1958* | 384 | non-ribosomal peptide synthase:amino acid adenylation; pyoverdine sidechain peptide synthetase II, D-Asp-L-Thr component | 782.943 | 569.145 | 0.460 | **1.376** | 5.833 | **5.45E-09** | 2.11E-07 | 1.92E-07 |
| ***pvdK*** | *Psyr_1959* | 384 | amino acid adenylation; pyoverdine sidechain peptide synthetase III, L-Thr-L-Ser component | 809.766 | 593.043 | 0.450 | **1.366** | 5.804 | **6.46E-09** | 2.45E-07 | 2.23E-07 |
| ***modA*** | *Psyr_2756* | 536 | molybdenum ABC transporter periplasmic-binding protein | 319.473 | 234.503 | 0.446 | **1.363** | 3.620 | **0.000294** | 0.0050098 | 0.004565 |
|  | *Psyr_2433* |  | hypothetical | 381.893 | 280.616 | 0.445 | **1.361** | 3.946 | **7.96E-05** | 0.0015176 | 0.001383 |
|  | *Psyr_1973* |  | aromatic amino acid aminotransferase; leucine biosynthesis | 310.323 | 228.120 | 0.444 | **1.361** | 3.552 | **0.000381** | 0.0063102 | 0.00575 |
| ***pvdD*** | *Psyr_1960* | 384 | non-ribosomal peptide synthase:amino acid adenylation; pyoverdine sidechain peptide synthetase IV, D-Asp-L-Ser component | 656.505 | 483.789 | 0.441 | **1.357** | 5.130 | **2.90E-07** | 8.80E-06 | 8.02E-06 |
| Table S1 Continued.  **Gene** | **Locus Tag** | **Op^a^** | **Function** | **RPKM *P.s.s.* B728a^b^** | **RPKM *P.s.s.* B728a *ΔacsS*^b^** | **log2 (Fold_change) normalized^d^** | **Fold Change^c^** | **z-score^d^** | **p-value^d^** | **q-value^d^** | **q-value^d^** |
|  | *Psyr_0133* |  | helix-turn-helix, Fis-type | 356.739 | 264.980 | 0.429 | **1.347** | 3.690 | **0.000224** | 0.0039599 | 0.003608 |
|  | *Psyr_4291* |  | hypothetical | 330.850 | 245.932 | 0.428 | **1.346** | 3.545 | **0.000392** | 0.0064631 | 0.00589 |
|  | *Psyr_2863* |  | feruloyl esterase; tannase precursor | 509.129 | 380.372 | 0.421 | **1.339** | 4.329 | **1.50E-05** | 0.0003567 | 0.000325 |
|  | *Psyr_2107* |  | ribosome modulation factor | 5804.014 | 4345.777 | 0.418 | **1.336** | 14.568 | **4.50E-48** | 1.46E-45 | 1.33E-45 |
| ***sodA*** | *Psyr_4152* | 810 | superoxide dismutase | 974.295 | 729.576 | 0.418 | **1.336** | 5.946 | **2.74E-09** | 1.14E-07 | 1.04E-07 |
|  | *Psyr_2145* | 424 | hypothetical | 309.444 | 232.489 | 0.413 | **1.331** | 3.314 | **0.000919** | 0.0137119 | 0.012495 |
| ***fliD*** | *Psyr_3464* | 667 | flagellar hook-associated protein 2, N-terminal:flagellar hook-associated 2, C-terminal:flagellin hook IN | 2177.497 | 1643.860 | 0.406 | **1.325** | 8.665 | **4.50E-18** | 3.83E-16 | 3.49E-16 |
|  | *Psyr_2646* |  | radical SAM family protein | 415.402 | 316.454 | 0.393 | **1.313** | 3.667 | **0.0002458** | 0.0042651 | 0.003887 |
|  | *Psyr_3845* |  | extracellular ligand-binding receptor | 549.911 | 425.465 | 0.370 | **1.293** | 3.994 | **6.49E-05** | 0.0012747 | 0.001162 |
| ***map1*** | *Psyr_2146* | 424 | peptidase M24A | 396.677 | 307.525 | 0.368 | **1.290** | 3.367 | **0.000759** | 0.0115144 | 0.010492 |
|  | *Psyr_0799* |  | fimbrial protein pilin, Type IV pilus | 1415.710 | 1098.756 | 0.366 | **1.289** | 6.340 | **2.30E-10** | 1.11E-08 | 1.02E-08 |
| ***fptA*** | *Psyr_1962* |  | TonB-dependent siderophore receptor | 1695.411 | 1384.836 | 0.292 | **1.224** | 5.612 | **1.99E-08** | 7.19E-07 | 6.55E-07 |
|  | *Psyr_0457* | 88 | hypothetical | 1555.410 | 1278.035 | 0.284 | **1.217** | 5.226 | **1.74E-07** | 5.30E-06 | 4.83E-06 |
| ***tssD*** | *Psyr_4965* |  | hypothetical; Hcp1 family type VI secretion system effector | 3903.616 | 3241.573 | 0.268 | **1.204** | 7.868 | **3.61E-15** | 2.46E-13 | 2.24E-13 |
| ***mucA*** | *Psyr_3957* | 768 | anti sigma-E protein RseA, N-terminal:anti sigma-E protein RseA, C-terminal | 1419.828 | 1198.843 | 0.244 | **1.185** | 4.330 | **1.49E-05** | 0.0003566 | 0.000325 |
| ***rpoS*** | *Psyr_1374* | 280 | RNA polymerase sigma factor RpoS | 1405.768 | 1195.902 | 0.234 | **1.176** | 4.125 | **3.70E-05** | 0.0007866 | 0.000717 |
| ***sucC*** | *Psyr_2012* | 397 | succinyl-CoA synthetase subunit beta; TCA cylce | 1312.132 | 1540.080 | -0.231 | **-1.174** | -4.271 | **1.95E-05** | 0.0004431 | 0.000404 |
|  | *Psyr_1117* |  | carbohydrate-selective porin OprB | 1093.614 | 1335.745 | -0.288 | **-1.221** | -4.917 | **8.78E-07** | 2.49E-05 | 2.27E-05 |
|  | *Psyr_3074* | 597 | hemolysin-type calcium-binding region:haemolysin-type calcium binding related | 600.837 | 755.218 | -0.330 | **-1.257** | -4.196 | **2.72E-05** | 0.0005971 | 0.000544 |
| ***atpD*** | *Psyr_5124* | 1005 | F0F1 ATP synthase subunit delta | 497.956 | 638.218 | -0.358 | **-1.281** | -4.166 | **3.11E-05** | 0.0006742 | 0.000614 |
| ***hfq*** | *Psyr_0572* | 109 | RNA-binding protein Hfq | 357.721 | 468.908 | -0.390 | **-1.311** | -3.872 | **0.000108** | 0.0019939 | 0.001817 |
|  | *Psyr_2005* | 395 | succinate dehydrogenase, cytochrome b subunit | 314.892 | 413.900 | -0.394 | **-1.314** | -3.672 | **0.000240** | 0.004205 | 0.003832 |
| Table S1 Continued.  **Gene** | **Locus Tag** | **Op^a^** | **Function** | **RPKM *P.s.s.* B728a^b^** | **RPKM *P.s.s.* B728a *ΔacsS*^b^** | **log2 (Fold_change) normalized^d^** | **Fold Change^c^** | **z-score^d^** | **p-value^d^** | **q-value^d^** | **q-value^d^** |
|  | *Psyr_3163* |  | serralysin | 2461.561 | 3273.277 | -0.411 | **-1.330** | -10.755 | **5.60E-27** | 8.55E-25 | 7.79E-25 |
|  | *Psyr_4400* | 854 | acetyl-CoA carboxylase biotin carboxyl carrier protein subunit | 230.288 | 306.815 | -0.414 | **-1.332** | -3.306 | **0.000944** | 0.0140061 | 0.012763 |
|  | *Psyr_3216* |  | hypothetical | 1805.100 | 2415.789 | -0.420 | **-1.338** | -9.427 | **4.23E-21** | 4.48E-19 | 4.08E-19 |
|  | *Psyr_2006* | 395 | succinate dehydrogenase, hydrophobic membrane anchor protein | 518.719 | 702.690 | -0.438 | **-1.354** | -5.274 | **1.34E-07** | 4.23E-06 | 3.85E-06 |
| ***atpB*** | *Psyr_5125* | 1005 | F0F1 ATP synthase subunit B | 351.420 | 476.642 | -0.439 | **-1.356** | -4.359 | **1.31E-05** | 0.0003153 | 0.000287 |
| ***fusA*** | *Psyr_4551* | 883 | elongation factor G | 1773.082 | 2439.396 | -0.460 | **-1.376** | -10.300 | **7.07E-25** | 9.65E-23 | 8.79E-23 |
| ***pvtK*** | *Psyr_4029* |  | pyruvate kinase | 174.154 | 242.130 | -0.475 | **-1.390** | -3.338 | **0.000844** | 0.0126546 | 0.011531 |
|  | *Psyr_2625* | 517 | putative lipoprotein | 203.912 | 284.110 | -0.478 | **-1.393** | -3.637 | **0.000275** | 0.0047314 | 0.004311 |
| ***atpA*** | *Psyr_5123* | 1005 | F0F1 ATP synthase subunit alpha | 264.965 | 375.652 | -0.503 | **-1.418** | -4.383 | **1.17E-05** | 0.0002876 | 0.000262 |
|  | *Psyr_2013* | 397 | succinyl-CoA synthetase subunit alpha; TCA cycle | 490.651 | 699.452 | -0.511 | **-1.425** | -6.068 | **1.29E-09** | 5.59E-08 | 5.10E-08 |
|  | *Psyr_2990* |  | hypothetical | 491.467 | 704.666 | -0.520 | **-1.434** | -6.181 | **6.37E-10** | 2.85E-08 | 2.60E-08 |
|  | *Psyr_5027* |  | histone-like DNA-binding protein | 158.550 | 235.689 | -0.572 | **-1.486** | -3.896 | **9.76E-05** | 0.0018084 | 0.001648 |
|  | *Psyr_0749* |  | AMP-dependent synthetase and ligase | 226.820 | 340.747 | -0.587 | **-1.502** | -4.797 | **1.61E-06** | 4.39E-05 | 4.00E-05 |
| ***tig*** | *Psyr_1746* | 357 | trigger factor; protease | 104.723 | 159.068 | -0.603 | **-1.519** | -3.357 | **0.000788** | 0.0118496 | 0.010798 |
| ***tsf*** | *Psyr_1344* |  | elongation factor Ts | 355.502 | 547.074 | -0.622 | **-1.539** | -6.401 | **1.55E-10** | 7.65E-09 | 6.97E-09 |
|  | *Psyr_0583* | 113 | hypothetical; predicted membrane protein | 264.033 | 407.290 | -0.625 | **-1.542** | -5.550 | **2.86E-08** | 1.01E-06 | 9.21E-07 |
| ***fabG-3*** | *Psyr_1647* | 335 | Lipid metabolism | 225.165 | 353.290 | -0.650 | **-1.569** | -5.348 | **8.87E-08** | 2.93E-06 | 2.67E-06 |
|  | *Psyr_0111* | 26 | hypothetical | 142.278 | 224.293 | -0.656 | **-1.576** | -4.301 | **1.70E-05** | 0.0003978 | 0.000362 |
| ***tpiA*** | *Psyr_4184* | 818 | triosephosphate isomerase; gluconeogenesis | 104.499 | 165.287 | -0.661 | **-1.581** | -3.716 | **0.000202** | 0.0035873 | 0.003269 |
| ***atpC*** | *Psyr_5122* | 1005 | F0F1 ATP synthase subunit gamma | 160.620 | 254.416 | -0.663 | **-1.584** | -4.623 | **3.78E-06** | 9.86E-05 | 8.99E-05 |
| ***pyrH*** | *Psyr_1345* | 274 | uridylate kinase; pyrimidine biosynthesis | 167.698 | 265.811 | -0.664 | **-1.585** | -4.732 | **2.23E-06** | 6.02E-05 | 5.48E-05 |
| ***atpD*** | *Psyr_5121* | 1005 | F0F1 ATP synthase subunit beta | 204.178 | 324.702 | -0.669 | **-1.590** | -5.263 | **1.42E-07** | 4.46E-06 | 4.05E-06 |
| Table S1 Continued.  **Gene** | **Locus Tag** | **Op^a^** | **Function** | **RPKM *P.s.s.* B728a^b^** | **RPKM *P.s.s.* B728a *ΔacsS*^b^** | **log2 (Fold_change) normalized^d^** | **Fold Change^c^** | **z-score^d^** | **p-value^d^** | **q-value^d^** | **q-value^d^** |
|  | *Psyr_4247* |  | ornithine decarboxylase | 211.504 | 337.714 | -0.675 | **-1.596** | -5.409 | **6.35E-08** | 2.14E-06 | 1.95E-06 |
| ***aroQ*** | *Psyr_4399* | 854 | 3-dehydroquinate dehydratase; phenylalanine/ tyrosine biosynthesis | 116.879 | 186.845 | -0.677 | **-1.598** | -4.032 | **5.54E-05** | 0.0011049 | 0.001007 |
|  | *Psyr_4987* | 976 | hypothetical | 146.727 | 235.365 | -0.682 | **-1.604** | -4.554 | **5.26E-06** | 0.0001337 | 0.000122 |
|  | *Psyr_0038* |  | hypothetical | 127.679 | 205.740 | -0.688 | **-1.611** | -4.294 | **1.76E-05** | 0.0004067 | 0.000371 |
| ***ppa-1*** | *Psyr_0624* |  | Energy production and conversion; TCA cycle | 87.623 | 141.251 | -0.689 | **-1.612** | -3.560 | **0.0003704** | 0.006179 | 0.005631 |
|  | *Psyr_0187* |  | hypothetical | 136.887 | 222.061 | -0.698 | **-1.622** | -4.516 | **6.30E-06** | 0.0001594 | 0.000145 |
| ***gltA*** | *Psyr_2004* |  | type II citrate synthase; TCA cycle | 190.068 | 310.474 | -0.708 | **-1.633** | -5.407 | **6.40E-08** | 2.14E-06 | 1.95E-06 |
| ***atpC*** | *Psyr_5126* | 1005 | F0F1 ATP synthase subunit C | 123.648 | 207.276 | -0.745 | **-1.676** | -4.621 | **3.82E-06** | 9.90E-05 | 9.02E-05 |
|  | *Psyr_0188* |  | hypothetical | 158.789 | 266.692 | -0.748 | **-1.679** | -5.259 | **1.45E-07** | 4.50E-06 | 4.10E-06 |
| **acpP** | Psyr_1648 |  | Phospholipid and fatty acid metabolism | 207.575 | 358.899 | -0.790 | **-1.729** | -6.396 | **1.60E-10** | 7.81E-09 | 7.12E-09 |
|  | *Psyr_1375* |  | 4Fe-4S ferredoxin, iron-sulfur binding | 55.158 | 98.752 | -0.840 | **-1.790** | -3.537 | **0.0004046** | 0.0066427 | 0.006053 |
|  | *Psyr_4175* |  | transport-associated protein | 51.899 | 95.297 | -0.876 | **-1.836** | -3.603 | **0.000314** | 0.0053212 | 0.004849 |
|  | *Psyr_2134* |  | hypothetical; putative lipoprotein | 137.329 | 256.502 | -0.901 | **-1.867** | -6.051 | **1.44E-09** | 6.16E-08 | 5.61E-08 |
| ***minE*** | *Psyr_1611* | 327 | cell division topological specificity factor MinE | 102.178 | 192.510 | -0.914 | **-1.884** | -5.304 | **1.14E-07** | 3.64E-06 | 3.30E-06 |
|  | *Psyr_5135* | 1008 | hypothetical | 112.706 | 213.082 | -0.919 | **-1.890** | -5.606 | **2.08E-08** | 7.43E-07 | 6.77E-07 |
| **rnpA** | *Psyr_5136* | 1008 | (Translation) | 82.164 | 159.413 | -0.956 | **-1.940** | -5.013 | **5.36E-07** | 1.58E-05 | 1.44E-05 |
| ***groEL*** | *Psyr_4072* | 794 | chaperonin GroEL | 280.605 | 546.041 | -0.960 | **-1.946** | -9.314 | **1.23E-20** | 1.25E-18 | 1.14E-18 |
| **ddl** | Psyr_3919 | 759 | Cell wall / LPS / capsule | 37.014 | 72.246 | -0.965 | **-1.952** | -3.400 | **0.000674** | 0.0102863 | 0.009373 |
| ***syrG*** | *Psyr_2602* |  | LuxR regulatory protein; phytotoxin regulation | 99.965 | 197.019 | -0.979 | **-1.971** | -5.683 | **1.33E-08** | 4.88E-07 | 4.45E-07 |
|  | *Psyr_2228* | 441 | hypothetical; nickel transport system substrate-binding protein | 48.801 | 97.429 | -0.997 | **-1.996** | -4.059 | **4.93E-05** | 0.0009958 | 0.000905 |
|  | *Psyr_0031* |  | Alpha/beta hydrolase fold | 211.561 | 423.674 | -1.002 | **-2.002** | -8.497 | **1.95E-17** | 1.56E-15 | 1.42E-15 |
|  | *Psyr_2229* | 441 | TonB-dependent siderophore receptor | 51.008 | 102.178 | -1.002 | **-2.003** | -4.173 | **3.00E-05** | 0.0006547 | 0.000597 |
| ***hrpZ1*** | *Psyr_1193* | 242 | type III helper protein HrpZ1 | 53.732 | 107.669 | -1.003 | **-2.003** | -4.286 | **1.82E-05** | 0.0004185 | 0.000381 |
|  | *Psyr_0750* | 151 | hypothetical; Ubiquinone biosynthesis | 133.491 | 276.390 | -1.050 | **-2.070** | -7.132 | **9.91E-13** | 5.47E-11 | 4.99E-11 |
| **Gene**  Table S1 Continued. | **Locus Tag** | **Op^a^** | **Function** | **RPKM *P.s.s.* B728a^b^** | **RPKM *P.s.s.* B728a *ΔacsS*^b^** | **log2 (Fold_change) normalized^d^** | **Fold Change^c^** | **z-score^d^** | **p-value^d^** | **q-value^d^** | **q-value^d^** |
| ***syrF*** | *Psyr_2607* | 512 | regulatory protein, LuxR; phytotoxin regulation | 31.354 | 65.354 | -1.059 | **-2.084** | -3.494 | **0.000476** | 0.00768 | 0.006998 |
| ***rpppK*** | *Psyr_0944* | 196 | ribose-phosphate pyrophosphokinase; Histidine biosynthesis | 72.518 | 151.440 | -1.062 | **-2.088** | -5.329 | **9.85E-08** | 3.24E-06 | 2.95E-06 |
|  | *Psyr_2366* | 466 | ABC transporter | 30.408 | 65.818 | -1.114 | **-2.164** | -3.651 | **0.000260** | 0.0044964 | 0.004097 |
|  | *Psyr_4652* | 912 | colicin immunity protein/pyocin immunity protein | 26.022 | 57.278 | -1.138 | **-2.201** | -3.466 | **0.000528** | 0.0084127 | 0.007666 |
|  | *Psyr_1643* | 334 | hypothetical | 106.287 | 234.512 | -1.141 | **-2.206** | -7.030 | **2.06E-12** | 1.11E-10 | 1.01E-10 |
|  | *Psyr_2497* |  | putative lipoprotein | 50.202 | 111.089 | -1.146 | **-2.212** | -4.853 | **1.22E-06** | 3.40E-05 | 3.09E-05 |
|  | *Psyr_1592* |  | hypothetical | 41.514 | 91.963 | -1.147 | **-2.215** | -4.420 | **9.87E-06** | 0.000245 | 0.000223 |
|  | *Psyr_4031* |  | Fe-S type hydro-lyase tartrate/fumarate alpha region:Fe-S type hydro-lyase tartrate/fumarate beta region; TCA cycle | 28.275 | 62.884 | -1.153 | **-2.224** | -3.669 | **0.000243** | 0.0042312 | 0.003856 |
|  | *Psyr_3730* | 722 |  | 25.200 | 56.265 | -1.159 | **-2.232** | -3.485 | **0.000492** | 0.0079146 | 0.007212 |
| ***groES*** | *Psyr_4073* | 794 | co-chaperonin GroES | 149.979 | 335.233 | -1.160 | **-2.235** | -8.516 | **1.65E-17** | 1.34E-15 | 1.22E-15 |
|  | *Psyr_1084* |  | histone-like DNA-binding protein | 105.425 | 240.062 | -1.187 | **-2.277** | -7.339 | **2.16E-13** | 1.24E-11 | 1.13E-11 |
|  | *Psyr_3028* |  | hypothetical | 29.851 | 68.451 | -1.197 | **-2.293** | -3.945 | **7.99E-05** | 0.0015177 | 0.001383 |
| ***hrpA2*** | *Psyr_1192* | 242 | type III helper protein HrpA2 | 31.668 | 72.864 | -1.202 | **-2.300** | -4.083 | **4.44E-05** | 0.0009112 | 0.00083 |
|  | *Psyr_0260* |  | hypothetical | 107.422 | 249.223 | -1.214 | **-2.320** | -7.612 | **2.71E-14** | 1.63E-12 | 1.49E-12 |
|  | *Psyr_5134* | 1008 | putative inner membrane protein translocase component YidC | 33.858 | 80.781 | -1.254 | **-2.385** | -4.446 | **8.75E-06** | 0.0002194 | 0.0002 |
|  | *Psyr_1471* |  | hypothetical | 290.811 | 705.266 | -1.278 | **-2.425** | -13.333 | **1.48E-40** | 3.84E-38 | 3.50E-38 |
| ***secG*** | *Psyr_4183* | 818 | preprotein translocase subunit SecG | 46.030 | 111.772 | -1.280 | **-2.428** | -5.312 | **1.08E-07** | 3.49E-06 | 3.18E-06 |
|  | *Psyr_2367* | 466 | hypothetical; predicted ABC transporter | 23.038 | 57.643 | -1.323 | **-2.502** | -3.914 | **9.07E-05** | 0.0016925 | 0.001538 |
|  | *Psyr_0835* |  | predicted RNA polymerase-binding protein DksA | 124.225 | 313.707 | -1.336 | **-2.525** | -9.203 | **3.47E-20** | 3.40E-18 | 3.10E-18 |
|  | *Psyr_0309* | 61 | colicin immunity protein/pyocin immunity protein | 41.603 | 105.563 | -1.343 | **-2.537** | -5.359 | **8.36E-08** | 2.78E-06 | 2.53E-06 |
|  | *Psyr_2156* | 426 | D-ribose pyranase | 19.383 | 49.493 | -1.352 | **-2.553** | -3.688 | **0.000225** | 0.0039674 | 0.003615 |
| ***syrB2*** | *Psyr_2610* | 513 | chlorinating enzyme; syringomycin biosynthesis | 66.236 | 187.275 | -1.499 | **-2.827** | -7.756 | **8.80E-15** | 5.78E-13 | 5.27E-13 |
| Table S1 Continued.  **Gene** | **Locus Tag** | **Op^a^** | **Function** | **RPKM *P.s.s.* B728a^b^** | **RPKM *P.s.s.* B728a *ΔacsS*^b^** | **log2 (Fold_change) normalized^d^** | **Fold Change^c^** | **z-score^d^** | **p-value^d^** | **q-value^d^** | **q-value^d^** |
| ***minC*** | *Psyr_1613* |  | septum formation inhibitor | 13.073 | 37.054 | -1.503 | **-2.834** | -3.456 | **0.000549** | 0.0086863 | 0.007915 |
|  | *Psyr_5074* | 996 | hypothetical | 42.333 | 121.070 | -1.516 | **-2.859** | -6.286 | **3.25E-10** | 1.55E-08 | 1.41E-08 |
|  | *Psyr_5102* | 1001 | ABC transporter | 12.205 | 35.977 | -1.559 | **-2.947** | -3.499 | **0.000467** | 0.0075584 | 0.006888 |
|  | *Psyr_5103* | 1001 | periplasmic binding protein | 11.591 | 35.076 | -1.597 | **-3.025** | -3.515 | **0.000439** | 0.0071455 | 0.006511 |
|  | *Psyr_2270* | 449 | hexapaptide repeat-containing transferase | 62.224 | 188.762 | -1.601 | **-3.033** | -8.169 | **3.12E-16** | 2.35E-14 | 2.14E-14 |
|  | *Psyr_2269* | 449 | putative GTP cyclohydrolase | 30.418 | 93.506 | -1.620 | **-3.074** | -5.798 | **6.70E-09** | 2.52E-07 | 2.29E-07 |
|  | *Psyr_3633* |  | threonine/serine transporter | 30.581 | 94.046 | -1.620 | **-3.075** | -5.817 | **6.01E-09** | 2.29E-07 | 2.09E-07 |
| ***syrD*** | *Psyr_2613* | 514 | cyclic peptide transporter; phytotoxin transport | 29.064 | 93.249 | -1.682 | **-3.208** | -5.947 | **2.73E-09** | 1.14E-07 | 1.04E-07 |
| ***syrB1*** | *Psyr_2611* | 513 | amino acid adenylation; syringomycin biosynthesis | 76.948 | 253.075 | -1.717 | **-3.288** | -9.945 | **2.66E-23** | 3.37E-21 | 3.07E-21 |
|  | *Psyr_5075* | 996 | cobalamin synthesis protein/P47K:cobalamin synthesis protein/P47K | 58.437 | 195.136 | -1.739 | **-3.339** | -8.810 | **1.25E-18** | 1.14E-16 | 1.04E-16 |
|  | *Psyr_1470* |  | hypothetical | 113.971 | 389.551 | -1.773 | **-3.417** | -12.616 | **1.73E-36** | 3.75E-34 | 3.41E-34 |
|  | *Psyr_2368* | 466 | periplasmic solute binding protein, cation ABC transporter | 60.329 | 217.474 | -1.850 | **-3.604** | -9.704 | **2.91E-22** | 3.43E-20 | 3.13E-20 |
| ***syrP*** | *Psyr_2612* | 514 | syringomycin synthesis and transport | 25.163 | 94.087 | -1.902 | **-3.738** | -6.504 | **7.80E-11** | 3.89E-09 | 3.55E-09 |
|  | *Psyr_1469* | 299 | hypothetical | 71.956 | 271.399 | -1.915 | **-3.771** | -11.097 | **1.30E-28** | 2.18E-26 | 1.99E-26 |
|  | *Psyr_2271* |  | hypothetical | 15.886 | 64.945 | -2.031 | **-4.087** | -5.642 | **1.68E-08** | 6.08E-07 | 5.54E-07 |
|  | *Psyr_1466* |  | hypothetical | 5.726 | 23.628 | -2.045 | **-4.125** | -3.418 | **0.000631** | 0.0097194 | 0.008857 |
|  | *Psyr_1468* | 299 | hypothetical | 34.969 | 166.491 | -2.251 | **-4.760** | -9.638 | **5.52E-22** | 6.36E-20 | 5.80E-20 |
|  | *Psyr_0768* |  | hypotherical | 3.212 | 18.425 | -2.520 | **-5.735** | -3.426 | **0.000613** | 0.0094709 | 0.00863 |
| ***avrPto1*** | *Psyr_4919* |  | type III effector protein AvrPto1 | 5.092 | 32.131 | -2.657 | **-6.309** | -4.658 | **3.19E-06** | 8.40E-05 | 7.65E-05 |

^a^ Operon predictions were made using the database of prokaryotic operons (<http://csbl1.bmb.uga.edu>).

^b^ Reads Per Kilobase per Million Mapped Reads [31].

^c^ Fold change is reflected as *P. syringae* pv. syringae B728a in comparison to the *ΔacsS* deletion mutant; therefore, a positive fold change reflects a decreased level of gene expression in *P. syringae* pv. syringae B728a *ΔacsS*.

^d^ The differential gene expression (log2 normalized), p-values and q-values were generated using the R sequence package DEGseq [32].
